# A Novel and Critical Role for Oct4 as a Regulator of the Maternal-Embryonic Transition

**DOI:** 10.1371/journal.pone.0004109

**Published:** 2008-12-31

**Authors:** Kira Foygel, Bokyung Choi, Sunny Jun, Denise E. Leong, Alan Lee, Connie C. Wong, Elizabeth Zuo, Michael Eckart, Renee A. Reijo Pera, Wing H. Wong, Mylene W. M. Yao

**Affiliations:** 1 Department of Obstetrics and Gynecology, Stanford University School of Medicine, Stanford, California, United States of America; 2 Department of Applied Physics, School of Humanities and Sciences, Stanford University, Stanford, California, United States of America; 3 Center for Human Embryo and Embryonic Stem Cell Research and Education, Institute for Stem Cell Biology & Regenerative Medicine, Stanford University, Palo Alto, California, United States of America; 4 Protein and Nucleic Acid Facility, Beckman Center, Stanford University, Stanford, California, United States of America; 5 Department of Statistics, School of Humanities and Sciences, Stanford University, California, United States of America; University of the Western Cape, South Africa

## Abstract

**Background:**

Compared to the emerging embryonic stem cell (ESC) gene network, little is known about the dynamic gene network that directs reprogramming in the early embryo. We hypothesized that Oct4, an ESC pluripotency regulator that is also highly expressed at the 1- to 2-cell stages in embryos, may be a critical regulator of the earliest gene network in the embryo.

**Methodology/Principal Findings:**

Using antisense morpholino oligonucleotide (MO)-mediated gene knockdown, we show that Oct4 is required for development prior to the blastocyst stage. Specifically, Oct4 has a novel and critical role in regulating genes that encode transcriptional and post-transcriptional regulators as early as the 2-cell stage. Our data suggest that the key function of Oct4 may be to switch the developmental program from one that is predominantly regulated by post-transcriptional control to one that depends on the transcriptional network. Further, we propose to rank candidate genes quantitatively based on the inter-embryo variation in their differential expression in response to *Oct4* knockdown. Of over 30 genes analyzed according to this proposed paradigm, *Rest* and *Mta2*, both of which have established pluripotency functions in ESCs, were found to be the most tightly regulated by Oct4 at the 2-cell stage.

**Conclusions/Significance:**

We show that the Oct4-regulated gene set at the 1- to 2-cell stages of early embryo development is large and distinct from its established network in ESCs. Further, our experimental approach can be applied to dissect the gene regulatory network of Oct4 and other pluripotency regulators to deconstruct the dynamic developmental program in the early embryo.

## Introduction

The early mammalian embryo, formed by the fusion of the highly differentiated egg and sperm, undergoes dramatic reprogramming. Totipotency or pluripotency is presumed to be established in blastomeres, followed by the first lineage-specific differentiation into trophectoderm and the inner cell mass (ICM) in the early blastocyst [1]. The developing fetus and embryonic stem cell (ESC) lines are derived from the ICM, so understanding early mammalian embryo development is critical to research on human diseases, and to the generation of pluripotent ESCs for therapeutic use [2]–[6]. Hence, determining the role of ESC regulators of self-renewal and pluripotency in the context of the early embryo may provide opportunities to better understand embryo development and ESC biology. (For our purposes here, the early embryo encompasses developmental stages that follow fertilization and precede blastocyst formation.)

Reprogramming in the early embryo is concurrent with massive degradation of maternal transcripts, and waves of embryonic activation that occur at the 1- to 2-cell, 4- to 8-cell (hereafter, multicell refers to stages between, but not including, 4-cell and morula), and morula to blastocyst stages [7]–[9]. However, the dynamic gene regulatory network that directs reprogramming has remained elusive. We approached this dynamic gene network by investigating the function of *Oct4* (also known as *Pou5f1*). *Oct4* expression is restricted to pluripotent cell types, and the level of Oct4 protein can direct lineage-specific differentiation in ESCs [10]–[13]. Despite rapid degradation of maternal *Oct4* transcripts starting at the 2-cell stage [10], [12], maternal and embryonic *Oct4* transcripts may transiently coexist. Consequently, Oct4 function specific to the maternal-embryonic transition cannot be addressed by *Oct4^−/−^* mice (which have defective ICM expansion) [10], conditional deletion of the maternal allele [supporting information (SI) Fig. S1], or studies using small interfering RNA (siRNA) [14], [15]; sufficiently rapid knockdown of both maternal and embryonic transcripts is unlikely to be possible. For example, RNAi-mediated *Oct4* knockdown resulted in development past the multi-cell and morula stages to a blastocyst-like state comprising giant trophoblasts and non-Oct4-expressing cells in the usual location of the ICM [15]. However, the role of Oct4 during the early cleavage stages, prior to the formation of the ICM, has not been investigated.

## Results

### Morpholino-mediated Gene Knockdown

**Figure 1:**
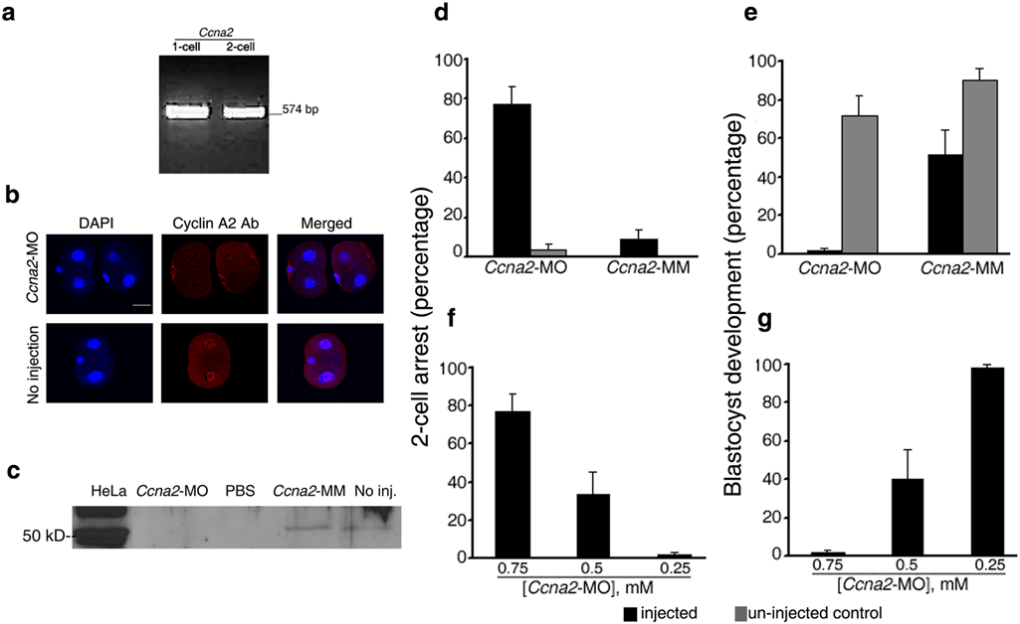
Translational block of cyclin A2 by *Ccna2*-MO causes embryos to arrest at the 2-cell stage. (*A*) *Ccna2* expression in 1- to 2-cell embryos by RT-PCR. (*B*) Nuclear cyclin A2 localization was absent in 83.4±6.0% of *Ccna2*-MO-injected embryos but present in all uninjected embryos and embryos injected with a mismatch control (*Ccna2*-MM); p<0.01). (*C*) ∼50 kD cyclin A2 protein was not detected in *Ccna2*-MO-injected embryos. (PBS, phosphate buffered saline.) (*D*) *Ccna2*-MO induced higher rates of 2-cell stage arrest compared to controls (p<0.01). (*E*) Only 1.8±1.8% of *Ccna2*-MO-injected embryos reached blastocyst stage (p = 0.06 compared to *Ccna2*-MM). (*F*) The rates of 2-cell stage arrest decreased with the concentration of *Ccna2*-MO (p = 0.05 for 0.5 mM; p = 0.01 for 0.25 mM). (*G*) The rate of blastocyst development at were higher at 0.25 mM than at 0.75 mM (p<0.001). (All columns and error bars represent mean±s.e.m., respectively, from at least three independent sets of experiments. Scale bar 40 µm. See SI Table S1 for the targeted sequence of all MOs used, and SI Table S2 for the total number of embryos tested in each set of experiments.)

Here, we provide proof-of-concept of the efficiency and specificity of MO-mediated gene knockdown in the mouse embryo by testing the procedure on the *Ccna2* gene. We then report the novel role of Oct4 that was revealed by MO-mediated gene knockdown. *Ccna2*, the gene encoding cell cycle regulator cyclin A2, has been suggested as an important transcriptional regulator in embryonic genome activation [16], a critical developmental milestone at the 1- to 2-cell stages for which few clear mechanisms or regulators have emerged. Consistent with the literature, MO-mediated *Ccna2* knockdown decreased cyclin A2 protein expression. In addition, our results showed that cyclin A2 is required for development beyond the 2-cell stage (Fig. 1, *A–G*, SI Tables S1 and S2). MOs block translation of transcripts by steric hindrance in an efficient and gene-specific manner, which has been well established in zebrafish and other model organisms [17]–[20]. Most importantly, MOs mediate rapid knockdown of transcripts regardless of their maternal or embryonic origin, before activation of downstream genes can provide partial “rescue” of the phenotype.

### *Oct4* Knockdown at the 1- to 2-Cell Stages

**Figure 2:**
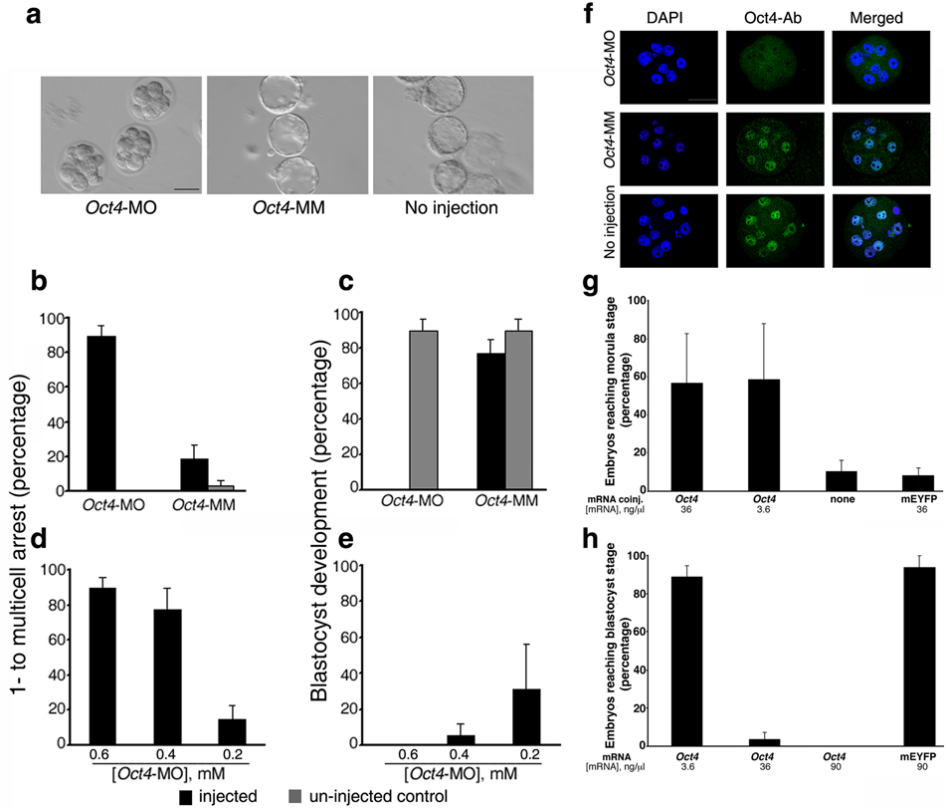
Oct4 is required for early embryo development prior to formation of the blastocyst. (*A*) *Oct4*-MO-injected embryos arrested at multicell stage, while uninjected and mismatch (*Oct4*-MM) controls reached blastocyst stage. (*B*) *Oct4* knockdown induced higher arrest rates at the 1- to multicell stages; p<0.01. (*C*) None of the *Oct4*-MO-injected embryos developed to blastocysts. (*D*) The rates of arrest at the 1- to multicell stages decreased with concentration of *Oct4*-MO (p<0.01). (*E*) There was a non-significant trend for higher rates of blastocyst development with decreasing concentrations of *Oct4*-MO. (*F*) Nuclear Oct4 expression is absent in *Oct4*-MO-injected embryos (top panel) but present in *Oct4*-MM-injected embryos and uninjected. The effect of *Oct4* knockdown could not be assessed by western blot because Oct4 protein was not detectable in pooled embryos by western blot (SI Fig. 6). (*G*) Compared to no coinjection or mEYFP mRNA co-injection, co-injection of 36 or 3.6 ng/µL of *Oct4* mRNA resulted in partial rescue of the *Oct4*-MO-induced phenotype by specifically decreasing the arrest rates at the 1- to multicell stages, which resulted in higher rates blastocyst development (p<0.01). (*H*) Overexpression of *Oct4* mRNA induced higher developmental arrest in a dose-dependent manner, such that blastocyst rates are significantly lower after injection of Oct4 mRNA at 36 or 90 ng/µL, compared to overexpression of mEYFP (p<0.01). (Scale bar = 40 µm).

**Figure 3:**
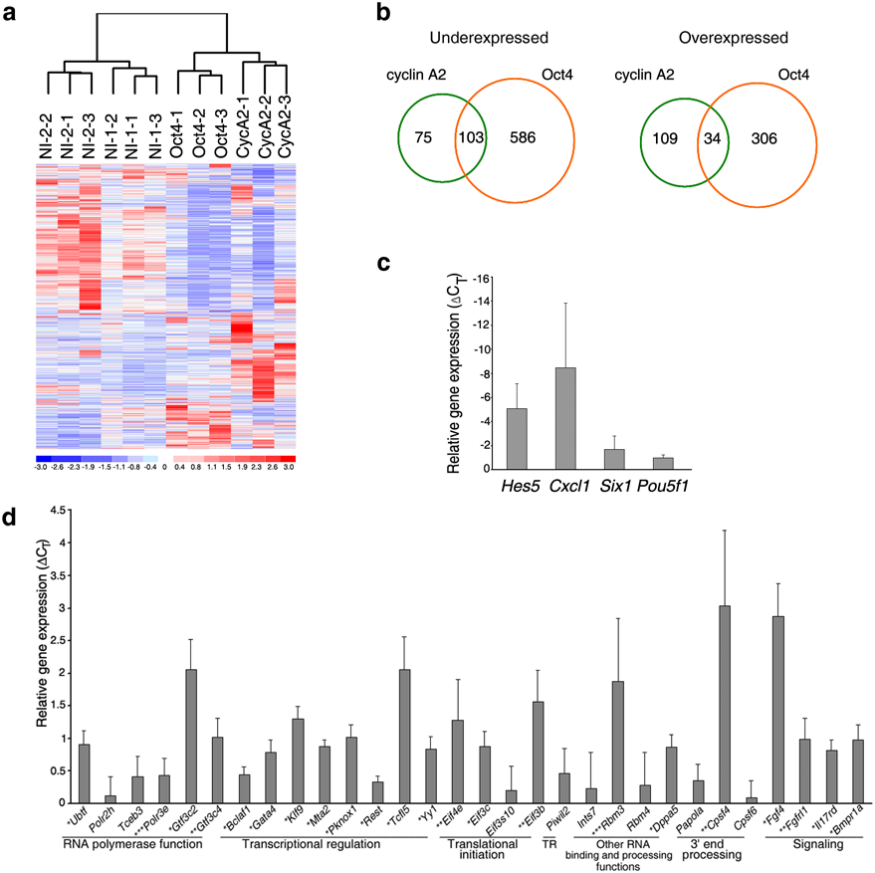
Gene regulation by Oct4. (*A*) Unsupervised clustering of 3 *Oct4* knockdown, 3 *Ccna2* knockdown, and 6 uninjected (NI) pooled embryo samples, and increased (red) and decreased (blue) gene expression. Scale is standard deviation. (*B*) Intersection of differentially expressed genes in *Oct4* and *Ccna2* knockdown embryos. Numbers indicate genes, not Affymetrix probesets. (*C–D*) Relative expression of *Oct4* knockdown by single-embryo q-PCR for (*C*) overexpressed genes (p<0.05 for *Hes5*) and (*D*) downregulated genes (*p<<0.001, **p<0.05, ***p<0.1). TR translational repression. Error bars indicate s.e.m. See SI Table S13 for a list of all the Taqman gene assays used for q-PCR experiments.

By combining MO-mediated gene knockdown with global gene expression profiling and single-embryo level quantitative RT-PCR (q-PCR), we determined the influence of Oct4 on gene expression, and analyzed the Oct4-regulated gene network in the early embryo (Figs. 2 and 3; SI Fig. S2). Consistent with the literature [12], we confirmed *Oct4* gene expression at the 1-cell stage (SI Fig. S3). After 1-cell embryos were microinjected with 0.6 mM *Oct4*-MO, the rate of developmental arrest at the 1- to multicell stages was dramatically higher than that observed for uninjected and mismatch (*Oct4*-MM) controls (Fig. 2, *A* and *B*). Of embryos injected with *Oct4*-MO that reached the multicell stage, 86.8±8.3% arrested and did not form morulae, compared to 10.5±10.5% embryos injected with *Oct4*-MM (p<0.01; data not shown). Most remarkably, none of the *Oct4*-MO-injected embryos developed to blastocysts, compared to relatively high blastocyst rates of *Oct4*-MM-injected and uninjected embryos (p<0.01; Fig. 2*C*). In contrast to the blastocyst-like stage that results after knock down of Oct4 with siRNA [15], embryos injected with *Oct4*-MO did not undergo further development or cell division after the multi-cell to morula stages. Further, the specificity of *Oct4*-MO is supported by the direct relationship between the phenotype severity and presumed “gene-dosage” as titrated by *Oct4*-MO concentration (Fig. 2, *D* and *E*). Oct4 protein expression was indeed reduced in *Oct4*-MO-injected embryos at the 4-cell (SI Fig. S4) and multi-cell stages (Fig. 2*F*, SI Fig. S5). However, *Oct4* knockdown could not be assessed by western blot (SI Fig. S6). Injection of another MO, targeting an intron-exon boundary in *Oct4*, confirmed that disruption of Oct4 function is detrimental to development before the blastocyst stage (SI Fig. S7).

### Partial Rescue and Overexpression by Microinjection of *Oct4* mRNA

The critical function of Oct4 at the 1- to 2-cell transition was embryo-autonomous. The effects of Oct4 knockdown could not be rescued by media conditioned by uninjected embryos or the *in vivo* environment provided by transferring injected embryos to oviducts of appropriately timed surrogate mothers (data not shown). Co-injection of low (3.6 ng/µL) or high (36 ng/µL) concentrations of unaltered, full-length *Oct4* mRNA with 0.6 mM *Oct4*-MO resulted in a decrease in the percentage of embryos arresting at the 1- to multicell stages, compared to co-injection of control mRNA encoding a modified enhanced yellow fluorescent protein (mEYFP) (Fig. 2*G*). No embryos co-injected with *Oct4* mRNA arrested at the multicell stage, while 80.0±5.2% arrested after co-injection of mEYFP mRNA (p<0.01; data not shown). Thus, co-injection of *Oct4* mRNA, but not control mRNA, partially rescued the *Oct4*-MO-induced multicell stage arrest phenotype. The failure to achieve full rescue of the phenotype and the lack of a difference in developmental rates between the low and high concentrations of *Oct4* mRNA may be due to relative instability of the *in vitro* transcribed *Oct4* mRNA compared to *Oct4*-MO. Alternatively, *Oct4* overexpression may have adverse effects that challenge attempts to rescue the *Oct4* knockdown phenotype.

Hence, we next tested whether *Oct4* over-expression itself would interfere with development. Injection of 36 ng/µL and 90 µL/nL of *Oct4* mRNA resulted in 8 and 40 folds increase in *Oct4* transcript levels, respectively, while Oct4 protein localization remained nuclear on immunocytochemistry (data not shown). *Oct4* over-expression indeed induced developmental arrest in a dosage-dependent manner, while injection of comparable or greater amounts of mEYFP mRNA interfered minimally with blastocyst development (Fig. 2*H*). The “gene dosage” effect of *Oct4* RNA injection may be due to enhanced Oct4 functions in transcriptional regulation, or Oct4 over-production may allow non-specific promoter-binding, or extra Oct4 causes inappropriate sequestration and subsequent inactivation of co-factors. Collectively, these data definitively showed *Oct4* expression was required, and that its correct level was critical to early embryo development, just as pluripotency of ESCs depends on a precise range of *Oct4* expression levels [11], [21].

### Identification of Differentially-Expressed Genes in *Oct4* Knockdown

To dissect the mechanisms of Oct4 function, we compared the global gene expression profile of *Oct4* knockdown embryos to the effects of *Ccna2* knockdown and to uninjected controls at the mid-2-cell stage. The goal was to identify differential gene expression that occurred with the first major wave of embryonic genome activation at the mid-2-cell stage [7], [8] (SI Tables S3, S4, S5 and S6). This time point was carefully selected to identify early genetic changes in response to *Oct4* knockdown, to precede developmental or cell cycle arrest at the multi-cell stage. Analysis by an unsupervised algorithm showed that the embryo samples clustered according to the experimental conditions, which further supported the specific and non-random effects of gene knockdown (Fig. 3*A*). At an arbitrary threshold false discovery rate (FDR) of 0.05, the Oct4-regulated gene set was more than 3 times larger, overall, compared to the number of genes changing expression in response to cyclin A2 knockdown (Fig. 3*B*). The different sizes of the under-expressed versus over-expressed *Oct4*-knockdown gene sets suggested that *Oct4* may be predominantly activating rather than repressing transcription (Fig. 3*B*). Some of the Oct4 candidate target genes have previously been identified as putative Oct4 targets based on mESC chromatin immunoprecipitation (ChIP) data or genomic sequence analysis of Oct4-binding sites [22] (SI Table S7).

The list of cyclin A2-regulated genes was rich in genes encoding factors for chromatin modification and remodelling (p = 0.005), nucleotide metabolism (p = 0.01), and chromosome organization (p = 0.01; SI Tables S8, S9). Oct4-regulated genes were significantly enriched for translation (p = 1.1×10^−4^) and RNA processing functions (p = 3.0×10^−5^) (SI Tables S10, S11). Comparison of our data with published Oct4-regulated networks in mouse ESCs [22] indicated that *Oct4* showed distinct and specific post-transcriptional and translational regulatory functions mediated by its control of genes encoding subunits in eukaryotic translation initiation factors (Eif), including *Eif3c*, and *Eif3b*. Interestingly, these two Eif subunits are evolutionarily conserved from yeast to human, and are amongst the six subunits comprising the functional core of mammalian Eif3, the largest of the Eif complexes [23].

In addition to its embryo-specific function, Oct4 also controls the expression of *Dppa5*, as it does in ESCs (data not shown); Dppa5 is an embryo-, germ cell- and ESC-specific RNA-binding protein whose role in maternal-embryonic transition is not known [24], [25]. *Piwil2* (also known as *Mili*), whose protein product and its bound pi-RNAs are known for their role in regulating retrotransposons in the fully-grown mouse oocyte [26], also showed a trend of decreased expression upon *Oct4* knockdown, although it did not reach statistical significance. As importantly, at the 1- to 2-cell stages, there was no evidence that the requirement of Oct4 in controlling the expression levels of *Cdx2* or *Nanog* [1], [27]–[30] was replicated in the 1- to 2-cell stages (data not shown). Although *Sox2* showed a trend of being over-expressed in *Oct4* knockdown, rather than being down-regulated as in ESCs, this response did not reach statistical significance (data not shown). Collectively, our data indicated that Oct4 has a distinct and specific role in the maternal-embryonic transition, in controlling genes encoding post-transcriptional regulators, in addition to its conserved functions shared amongst pluripotent cell types.

### Maternal Transcript Degradation and Embryonic Genome Activation

In order to understand the role of Oct4 in reprogramming the early embryo, we examined its role in embryonic genome activation and maternal transcript degradation. Overall, Oct4 regulates gene expression pertinent to basic machinery required for the entire spectrum of gene regulation, including transcription involving all three RNA polymerases, translation, RNA processing such as regulation of polyadenylation, and mRNA degradation proteins (SI Table S12). High levels of mRNA from developmental genes, such as *Six1*, *Nestin*, and *Hoxa3*, indicated that Oct4 was required for their repression, while excessive levels of maternal transcripts that would normally be rapidly degraded, such as *Zar1* and *Nobox1*, indicated that Oct4 knockdown interfered with the mRNA degradation machinery. Thus, Oct4 has developmental stage- and cell-specific functions, and has an important role in the processes that mark maternal-embryonic transition.

### Single-Embryo Quantitative RT-PCR (q-PCR)

To further define the Oct4-regulated gene network, we selected 42 genes representing transcriptional, post-transcriptional and signalling functions for q-PCR assays. We analyzed RNA from single *Oct4*-MO-injected and control embryos and focused on genes that were under-expressed in *Oct4* knockdown. After removing data related to 3 genes for which there were technical difficulties, expression changes of 39 genes were appropriately measured based on analysis using a linear model (See Methods S1 in Supplementary Online Materials). Of those, 34 or ∼87% showed altered expression levels in *Oct4* knockdown in the expected directions (Fig. 3 *C* and *D*), while 5 genes, including *Sox2*, did not change (data not shown). 21 of the 34 genes, or ∼62%, showed statistically significant differential expression by q-PCR at p<0.05 or less, while injection of a control MO targeting the human globin gene, which would not be present in the mouse genome, did not alter expression of any of the genes assayed (data not shown). Thus, we have proven that Oct4 directly or indirectly regulates genes encoding a wide range of transcriptional and post-transcriptional regulators at the 1- to 2-cell stages.

### Quantitative Ranking of Candidate Genes in the Oct4-Regulated Early Embryo Network

**Figure 4:**
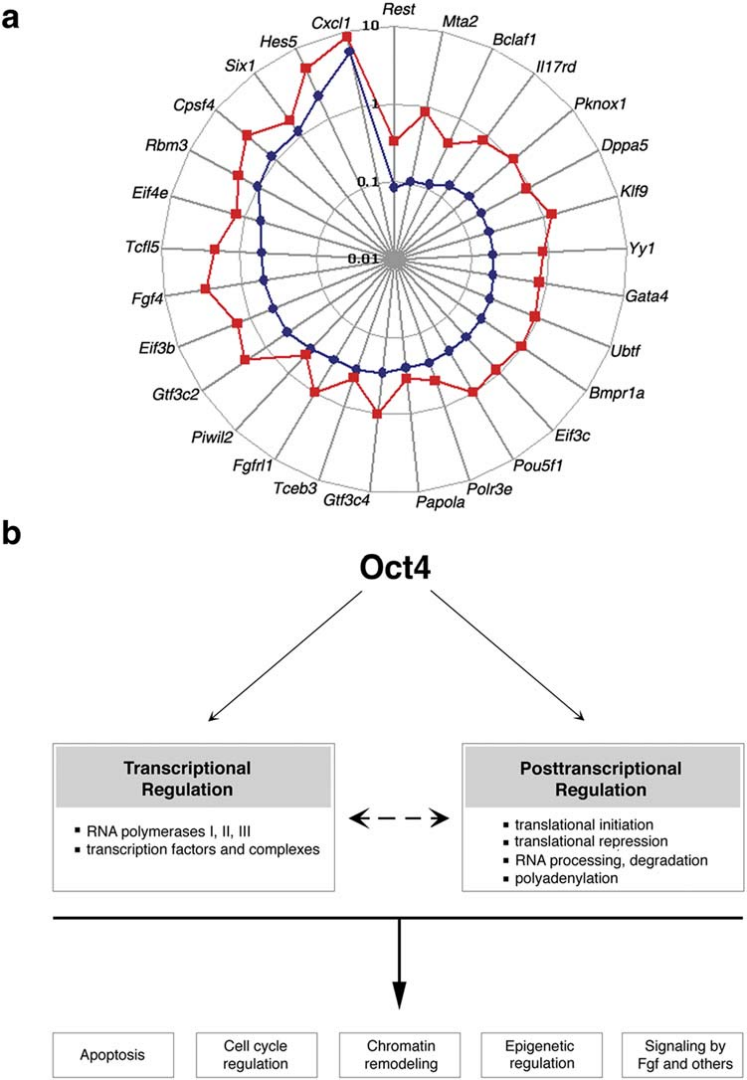
Models of Oct4 function at the 1- to 2-cell stage. (*A*) Genes whose Absolute (ΔC_T_) (red) is greater than its s.e.m. (blue), are ordered clockwise based on increasing s.e.m. (Log scale.) (*B*) Proposed regulation of various modules by Oct4 via transcriptional and post-transcriptional mechanisms.

Our single-embryo data allowed us to go beyond simply validating our gene chip data. Methods using samples comprised of pooled cells or embryos, generate relative gene expression that represents an average of all cells assayed, but they cannot discern between genes that are consistently differentially regulated versus those with a tendency towards stochastic changes; similarly, rare outlier embryos expressing unique transcriptomes are not recognized [31]–[33]. By analyzing quantitative expression data at the single-embryo level, we were able to make this discrimination. We presume genes whose relative expression is consistent amongst single embryos have a higher likelihood to be essential nodes in a gene regulatory network, which is expected to respond to perturbations in a consistent and predictable manner. The gene set was restricted to genes whose differential expression (represented by the difference in threshold cycles, ΔC_T_) ΔC_T_ is greater than expression differences amongst single embryos (represented by standard error of the mean, s.e.m.). We propose a hierarchy in the Oct4-regulated gene network in which 29 genes are ordered based on their increasing s.e.m., or inter-embryo variation and presumed decreasing biological significance in this network (Fig. 4*A*). Interestingly, *Rest*, which has critical pluripotency functions in ESCs [34], and *Mta2*, which encodes a member of the Nanog and Oct4-associated deacetylase complex (NODE) [35], were found to be the most tightly regulated by Oct4, based on the small inter-embryo variation in their differential expression in response to *Oct4* knockdown. Taken together, we have identified and ranked potential key nodes of this network in a quantitative fashion.

## Discussion

By exploiting our experimental strategy comprising MO-mediated gene knockdown, global gene expression analysis, and semi-quantitative gene expression analysis, simultaneous changes in relative expression levels can be correlated for an entire network of genes at the level of the single embryo to facilitate dissection of the regulatory network. We envision that these Oct4-regulated genes may serve as “portals” through which we can peer into and deconstruct the dynamic gene network that directs development and cell fate decisions, and that leads to the establishment of the ESC gene network. Further, the lack of impact of *Oct4* knockdown on the expression levels of *Cdx2*, *Nanog* and *Sox2* supports that the role of *Oct4* at the maternal-embryonic transition is distinct from its well-established functions in ESCs, and suggests that other pluripotency regulators may also have different roles in the early embryo.

Our data suggest that in the unique developmental context of maternal-embryonic transition, concomitant with massive mRNA degradation and dramatic reprogramming, Oct4 controls the expression of many transcriptional regulators. Oct4 also maintains the expression of many genes, such as *Eif3c*, *Papola*, *Eif3b*, *Eif4e*, *Rbm3* and *Cpsf4*, that are involved in the post-transcriptional control. Through its influence on both the transcriptional and post-transcriptional regulators, Oct4 can directly or indirectly affect many essential processes, such as chromatin remodelling, epigenetic regulation, apoptosis, cell cycle regulation, and signalling, during early developmental program (Fig. 4*B*).

Given the importance of post-transcriptional regulation in the transcriptionally silent mature oocyte, and the recapitulation of some of the blastocyst and ESC Oct4 functions in the early embryo–regulation of *Fgf4* [10], [36], *Klf* family member [37], *Dppa5* [22] expression–the dual function of Oct4 in post-transcriptional and transcriptional regulation at the maternal-embryonic transition suggests how stage-specific developmental requirements can be fulfilled by a conserved pattern of gene networks. In fact, Oct4 may serve not only as a link between the germ cell and embryonic developmental programs, but also as a switch from a post-transcriptionally-regulated program to one that depends on the transcriptional network. Future investigations of the precise mechanisms by which *Oct4* controls the early embryonic transcriptional and post-transcriptional programs will uncover important mechanisms of reprogramming in the early embryo, and perhaps ESCs.

## Materials and Methods

### Embryo culture

All procedures involving animals were performed under our active, Institutional Animal Care and Use Committee (IACUC) protocol #8315 entitled “*Developmental Regulation of the Mammalian Embryo*”, which was approved by the Administrative Panel on Laboratory Animal Care (APLAC) at Stanford University. 3–5 week old wild type F1 (C57BL6×DBA/2) females (Charles River) were superovulated by intraperitonial injections of 5 IU of pregnant mare's serum gonadotropin (Sigma) followed by 5 IU of human chorionic gonadotropin (Sigma) 48 hours later, and mated overnight with wild type males. Mice were sacrificed by cervical dislocation 17 hours after hCG injection, and 1-cell embryos were released from oviducts. Cumulus cells were removed by hyaluronidase (Sigma) treatment and pipetting. Pre-implantation embryos at the two pronuclei stage were recovered, pooled from 3–6 females in M2 media (Chemicon International), followed by immediate cytoplasmic microinjection and culture in Human Tubal Fluid with 10% serum supplement (In-Vitro Fertilization, Inc.) microdrops under mineral oil (Sigma) in mixed gas (90% nitrogen, 5% oxygen, 5% carbon dioxide; Praxair) at 37°C, and cultured at ten embryos per 20 µL drop.

### Microinjection of antisense morpholino oligonucleotides

25-nt, antisense morpholino oligonucleotides (MOs) that specifically target the 5′UTR or translational start site, or controls mismatched at 5 nts were purchased from Gene Tools, LLC. (See Supplementary Table 1 for sequence details). We had determined 0.6–0.75 mM to be the maximal concentration that would allow normal rates of blastocyst development (data not shown). Hence, unless otherwise specified, 5–10 pL of 0.75 mM *Ccna2*-MO (0.60 mM for *Oct4*-MO) was injected into the cytoplasm of each embryo on an inverted microscope (Olympus IX70) equipped with hydraulic micromanipulation system (IM300 Microinjector, Narishige, Japan). 10 uninjected control embryos were used in each experiment, which was performed at least three times. The mean percentage and standard error of the mean (mean±s.e.m.) of embryos progressing to, or arresting at, each developmental stage were calculated, and statistical significance was determined by calculating the p-value using 2-tailed Student's t-test.

See Supporting Information for detailed materials and methods.

### Control morpholino oligonucleotides

In each experiment, uninjected embryos and embryos injected with a control morpholino were tested in parallel with *Oct4*-MO-mediated knockdown. Overall, we used three different types of control morpholinos, but only one type of control morpholino was used in each experiment. 1) We used mismatch control morpholinos in the embryo phenotype experiments to control for the morpholino sequence, save 5 nucleotides. This mismatch control would have controlled for the microinjection, the presence of exogenous morpholino oligonucleotides in the cytoplasm, but did not control for the biological effects of having morpholino-bound transcripts in the cytoplasm. 2) In the gene chip experiments, we targeted cyclin A2 transcript with specific morpholinos. Here, the cyclin A2 experiments controlled for microinjection, the presence of oligonucleotides in the cytoplasm, and the biological effects of having morpholino-bound transcripts in the cytoplasm. (Note that it was not possible to control for having morpholino-bound *Oct4* transcripts without actually knocking down *Oct4*.) 3) In the single-embryo qPCR experiments, we used a control morpholino that was designed to specifically target the human globin (HG) gene promoter, which is not present in the mouse genome. We had tested this HG morpholino when establishing our methods and found that HG morpholino did not affect blastocyst developmental rates. Importantly, genes that were validated to be differentially-expressed between uninjected and *Oct4*-MO-injected embryos were also confirmed to show no differential expression between uninjected and *HG*-MO-injected embryos. Therefore, the single-embryo qPCR experiments were controlled for microinjection and the presence of morpholino oligonucleotides in the cytoplasm.

## Supporting Information

Methods S1 Detailed methods.
(0.05 MB DOC)

Figure S1 Limitation of conventional gene-targeting strategies for the study of gene function during the maternal-embryonic transition. Our overall goal is to address the specific functions of a given gene product at the maternal-embryonic transition in order to understand mechanisms that regulate mammalian embryo development at the earliest stages. In wild type (+/−) embryos, maternal transcripts are present before embryonic genome activation (EGA), while maternal and embryonic transcripts are present at maternal-embryonic transition stage, both resulting in production of a normal gene product (A). In homozygous null mutant (−/−) embryos generated from a mother that is heterozygous (+/−) for the null mutation, persistent maternal transcripts and/or proteins may “rescue” or delay the phenotype onset (B). In contrast, homozygous null mutant embryos generated from a homozygous mutant female, or a female with oocyte-specific gene deletion, the observed defects may reflect oocyte defects, rather than specific gene requirement in the early embryo (C). Therefore, these strategies do not address the precise roles of specific genes at the cusp of EGA or during EGA, when both maternal and early embryonic transcripts may be present simultaneously. Cytoplasmic microinjection of antisense morpholino oligonucleotides (MOs) into wild type embryo just at or before EGA results in specific translational block of both maternal and embryonic gene transcripts (D). Since MOs persist for at least a few cycles of cell division, gene-specific translational block is presumably effective until the morula-blastocyst stages (1–3). The absence of gene product during these developmental stages would reveal critical gene function and unmask early phenotypes that may not be detectable in conventional gene-targeting strategies by homologous recombination and transgenesis. While this model is well established in other species (2, 4, 5), it shifts the paradigm from investigating function of embryonic genes to that of gene products regardless of their maternal or embryonic origin. References. 1. Morcos PA (2007) Achieving targeted and quantifiable alteration of mRNA splicing with Morpholino oligos. Biochem Biophys Res Commun 358, 521–527. 2. Sumanas S & Larson JD (2002) Morpholino phosphorodiamidate oligonucleotides in zebrafish: a recipe for functional genomics? Brief Funct Genomic Proteomic 1, 239–256. 3. Summerton J, et al. (1997) Morpholino and phosphorothioate antisense oligomers compared in cell-free and in-cell systems. Antisense Nucleic Acid Drug Dev 7, 63–70. 4. Imai KS, Levine M, Satoh N, & Satou Y (2006) Regulatory blueprint for a chordate embryo. Science 312, 1183–1187. 5. Yamada L, et al. (2003) Morpholino-based gene knockdown screen of novel genes with developmental function in Ciona intestinalis. Development 130, 6485–6495.
(0.75 MB DOC)

Figure S2 Experimental strategy. Embryos at the 2 pronuclei (2PN) or 1-cell stage are collected from wild type matings, and injected with an antisense morpholino oligomer (MO) that has been designed to target a specific gene. MO binds to 5′ UTR or transcription start site and blocks translation by steric hindrance. Microinjected embryos and uninjected control embryos are cultured in vitro and observed for developmental phenotypes such as fragmentation, or arrest at the 2-cell, 4-cell, multicell, or morula stages. Theoretically, this strategy may uncover other phenotypes such as asymmetrical division, but we have not observed them in the genes that we have tested. If a gene-specific MO produces the same phenotype consistently, while the mismatch control MO allows normal development, then we validate knockdown of the gene of interest by immunocytochemistry and/or immunoblotting. Mechanism of gene function is further investigated by obtaining global gene expression profiles from injected and control embryos at the mid-2-cell stage (43 hours post-HCG). Candidate downstream genes are tested for differential expression, and gene function in the early embryo. It is expected that multiple iterations of this strategy to test functions of different transcriptional regulators and their downstream targets will help to deconstruct the gene regulatory network in the mouse embryo at the cusp of embryonic genome activation.
(0.77 MB DOC)

Figure S3 Oct4 expression in the mouse zygote by single embryo RT-PCR.
(0.11 MB TIF)

Figure S4 Decreased Oct4 expression was evident by the 4-cell stage in Oct4-MO-injected embryos. Oct4 signal was absent in embryos injected with Oct4-MO, but its nuclear localization was present in uninjected and mismatch controls. Scale bar 40 µm.
(10.94 MB TIF)

Figure S5 Decreased Oct4 expression at the multicell stage in Oct4-MO-injected embryos. Only 6.4±3.2% of Oct4-MO-injected embryos showed nuclear Oct4 signal, while 88.9±11.1% of Oct4-MM-injected embryos and 82.7±10.9% of uninjected control embryos showed unequivocal nuclear Oct4 expression at the multicell stage; p<0.05.
(10.82 MB TIF)

Figure S6 Oct4 knockdown could not be assessed by western blot. Anti-Oct4 antibody detected specific Oct4 band in F9 mouse embryonal carcinoma cell line, but not in the lane containing pooled protein lysate from 57 mouse blastocysts. In contrast, the band corresponding to RNA polymerase II (subunit A) was detectable in both F9 cells and mouse blastocysts. This figure is representative of experiments showing that 50–100 embryos at the 2-cell, multicell, and blastocyst stages did not have sufficient amounts of Oct4 protein for detection by western blot. (PBS, phosphate buffered saline)
(0.57 MB TIF)

Figure S7 Confirmation of the requirement of Oct4 in early embryo development by Oct4E4-MO, an antisense morpholino that targets the splice site of exon 4 of Oct4. a, sites targeted by the two morpholinos, Oct4-MO and Oct4E4-MO. Oct4-MO targets the 25 nucleotides starting at the ATG start site, while Oct4E4-MO targets the splice site at the intron (I)-exon (E) boundary of the 4th exon (E4). Removal of E4 is expected to result in a protein product that lacks the DNA-binding and activation domains (1). b, 64.6±19.9% of embryos injected with Oct4E4-MO, while none that were injected with the mismatch control, Oct4E4-MM, arrested at the 2-cell stage. c, Blastocyst development is severely compromised after injection of Oct4E4-MO compared to the mismatch control, Oct4E4-MM. Reference. 1. Morcos PA (2007) Achieving targeted and quantifiable alteration of mRNA splicing with Morpholino oligos. Biochem Biophys Res Commun 358, 521–527.
(2.33 MB TIF)

Table S1 Antisense morpholino oligonucleotides target gene-specific sequence in the 5′UTR and/or start site.
(0.06 MB PDF)

Table S2 Summary of the number of embryos tested and the number of experiments performed for each condition.
(0.07 MB PDF)

Table S3 Genes that have higher expression levels in Oct4-MO-injected compared to uninjected embryos.
(0.05 MB XLS)

Table S4 Genes that have lower expression levels in Oct4-MO-injected compared to uninjected embryos.
(0.11 MB XLS)

Table S5 Genes that have higher expression levels in Ccna2-MO-injected compared to uninjected embryos.
(0.03 MB XLS)

Table S6 Genes that have lower expression levels in Ccna2-MO-injected compared to uninjected embryos.
(0.04 MB XLS)

Table S7 Oct4 candidate target genes that have putative Oct4 binding sites based on genomic sequence analysis or mouse ESC chromatin precipitation data*. *Data were compared to those reported in: Zhou Q, Chipperfield H, Melton DA, & Wong WH (2007) A gene regulatory network in mouse embryonic stem cells. Proc Natl Acad Sci U S A 104, 16438–16443.
(0.02 MB PDF)

Table S8 Functional categories that were enriched in downregulated genes in the Ccna2 knockdown model.
(0.03 MB PDF)

Table S9 Functional categories that were enriched in upregulated genes in the Ccna2 knockdown model.
(0.01 MB PDF)

Table S10 Functional categories that were enriched in downregulated genes in the Oct4 knockdown model.
(0.02 MB PDF)

Table S11 Functional categories that were enriched in upregulated genes in the Oct4 knockdown model.
(0.01 MB PDF)

Table S12 Some candidate Oct4-regulated genes that function in transcription, translation, RNA processing, chromatin remodeling, signaling, apoptosis and the cell cycle.
(0.08 MB PDF)

Table S13 Gene-specific primers and Taqman® probes that were used in q-PCR experiments.
(0.04 MB PDF)
