# Supplementary material for: A Novel and Critical Role for Oct4 as a Regulator of the Maternal-Embryonic Transition
Source: PLoS One. 2008 Dec 31;3(12):e4109. doi: 10.1371/journal.pone.0004109 (PMC2614881; doi:10.1371/journal.pone.0004109)
Supplement: Methods S1 — Detailed methods. (0.05 MB DOC) [file pone.0004109.s001.doc]

**SUPPORTING INFORMATION**

**Materials and Methods**

**Immunoblot and immunocytochemistry.** Injected and control embryos were collected at 2-cell stage (43 hours post human chorionic gonadotropin (hCG) administration) and washed in PBS containing 3mg/mL polyvinylpyrrolidone (PVP). For immunoblot, injected and control embryos were lysed in RIPA buffer (50 mM Tris-HCl, pH7.5, 150 mM NaCl, 1 mM EDTA, 1% Nonident P-40, 2 mg/ml aprotonin, 2 mg/ml leupeptin, 1 mg/ml pepstain, and 20 mg/ml phenylmethylsulfonyl) containing a phosphatase inhibitor cocktail (Roche), boiled in Laemli buffer, and stored at -80°C until lysates from 75 embryos were collected for each condition. Samples were loaded and analyzed by electrophoresis on 10% Tris-HCl polyacrylamide gel, semi-dry transferred onto nitrocellulose membrane (Bio-Rad), blocked in 0.1% Tween-20-1% Casein TBS Blocking Solution (Bio-Rad), incubated overnight in 1:250 diluted primary rabbit polyclonal anti-cyclin A2 antibody (Santa Cruz Biotechnologies, sc-751) at 4°C and in 1:2000 diluted secondary donkey anti-rabbit horseradish peroxidase-linked antibody (Amersham, NA934V) for one hour at room temperature, and visualized using ECL Blotting Detection Reagent (Amersham). Immunocytochemistry was performed according to standard protocol. Briefly, embryos were fixed in 4% paraformaldehyde-PBS solution, permeabilized in 0.1% Triton X-PBS and treated with ImageIT FX Signal Enhancer solution (Invitrogen) at RT, incubated in 1:100 diluted primary antibody overnight at 4°C, in 1:10,000 diluted secondary antibody for one hour, followed by 3μM DAPI for 10 min., and were mounted in VectaShield Mounting Medium (Vector Laboratories, H-1000). Controls were performed in parallel with normal rabbit or mouse serum control. All antibodies were diluted in 1% BSA. Embryos were imaged by confocal microscopy using LSM 510 Confocal Laser Scanning Microscope or epifluorescence microscopy or the Axiovert 200 microscope equipped with an Axiocam digital camera (Zeiss) using fixed parameters and exposure times. Primary antibodies were purchased from Santa Cruz Biotechnologies (anti-cyclin A2 rabbit polyclonal (sc-751), normal rabbit IgG (sc-2027), mouse monoclonal anti-Oct4 (sc-5279), mouse monoclonal anti-RNA Polymerase II subunit a (sc-55492), and normal mouse IgG (sc-2025). Secondary antibodies were purchased from Molecular Probes (Alexa Fluor 594 goat anti-rabbit IgG (A-11012) and goat-anti mouse IgG (A-11001).

**mRNA synthesis by *in vitro* transcription*.*** Full-length mouse cDNA clone, Oct4-pSPORT (clone ID 30019896) (Open Biosystems), and a plasmid encoding the fluorescence mitotic biosensor, a modified enhanced yellow fluorescence protein (mEYFP), were sequence-verified, linearized by restriction enzyme digest, and used as templates. 5’ capped and polyadenylated mRNA transcripts were transcribed *in vitro* (mMessage and PolyA-Tail kits, Ambion), which were then quantitated by UV spectroscopy, and analyzed by electrophoresis to confirm size.

**RNA sample preparation for gene chip experiments.** Samples containing 20 pooled injected or control mouse embryos were washed through 3 drops of PBS/PVP and collected for total RNA extraction and isolation (Picopure Total RNA Isolation Kit, Molecular Devices Corp.), to yield 10 μL of total RNA. 5 μL of total RNA, or the equivalent of 10 mouse embryos, was subjected to two rounds of amplification (WT-Ovation Pico system, Nugen) according to manufacturer’s instructions. The quality of ssDNA resulting from the second round amplification was tested on the Bioanalyzer 2100 (Agilent), and a typical yield of 5-8 μg per sample was quantitated by the ND-1000 UV spectrophotometer (Nanodrop Technologies). Direct biotin labeling and fragmentation were performed (FL-Ovation cDNA Biotin Module (Nugen). Fragmented, labeled, ssDNA samples were submitted to Stanford University PAN Core Facility for hybridization to the GeneChip® Mouse Genome 430 2.0 Array (Affymetrix), and laser scanning.

**Statistical analysis for gene chip experiments*.*** Raw data from a total of 12 gene chips (3 *Ccna2*-MO and their uninjected controls, and 3 *Oct4-*MO and their uninjected controls) and were normalized by dChip (1). Unsupervised clustering analysis was performed for genes that have: 1) expression level greater than 500 Signal Intensity (SI) in at least 10 percent of the samples; 2) standard deviation to mean ratio > 0.4 and < 1000 across the samples. Subsequent analyses were not restricted to these criteria. The lists of differentially expressed genes and their ranking were generated by the method proposed by Johnson and Wong, which is based on fold change, logged fold change and unpaired t-statistic (Nicholas Johnson and W.H.W. (2007) Combining scientific and statistical significance in gene ranking. Unpublished.). Differential expression was defined by a threshold of 5 percent median false discovery rate (FDR) estimated from 300 random permutations across the samples. To find significantly enriched gene ontology (GO) terms (2), each gene list was mapped to Entrez gene identification numbers (NCBI) and tested by GOSTAT PACKAGE (3) against a set of “universe” genes defined by “P” call in at least 2 out of 3 treated samples or 4 out of 6 control samples by (MAS, Affymetrix). To estimate the FDR, 50 tests were performed with randomly generated lists of genes from the “universe” gene set, and the average number of enriched GO terms were calculated (2, 3). The cut off for p-value was chosen to reflect FDR of ~10 percent. Differentially expressed genes with possible Oct4-binding sites were identified by comparison with putative Oct4-regulated genes previously identified by Zhou *et. al*. (4), followed by mapping of probe sets to Refseq using the BIOCONDUCTOR ANNOT PACKAGE (5).

**RT-PCR, conventional quantitative PCR (q-PCR), and single embryo q-PCR analysis.** Lysis buffer was added to PBS/PVP washed embryos (Cells-to-cDNA kit, Ambion) and samples were treated with 1 μL of DNAse I. Reverse transcription (RT) was performed by using 1.0 μL of SuperScript III RT enzyme (200 U/μL) as per protocol (Invitrogen). Amplification of gene-specific product by TaqPolymerase High Fidelity Kit (Invitrogen) was performed on the thermocycler (Mastercycler gradient 5331, Eppendorf) as follows: 94.0°C for 2 min., 94.0°C for 15 sec., 60.0°C for 30 sec., 68.0°C for 45 sec, 68.0°C for 7 minutes for 50 cycles. (See Supplementary Table S8 for all primer sequence and TaqMan probes.) Single embryo qRT-PCR was performed using the Biomark 48.48 Dynamic Array system (Fluidigm, South San Francisco, CA). Single embryos were treated with acid tyrode, and collected in 10 μl reaction buffer, followed by preamplification as per manufacturer’s instructions (TaqMan Gene Expression Assay, Applied Biosystems; Table S8). Amplified cDNA was loaded onto a 48.48 Dynamic Array using the NanoFlex IFC controller (Fluidigm). Threshold cycle (C_T_) as a measurement of relative fluorescence intensity was extracted from the BioMark PCR analysis software (Fluidigm). All reactions were performed in duplicates or triplicates along with negative RT, PBS, and positive controls in at least three to five independent experiments. Data for each gene assayed were tested with a linear model (ANCOVA) in which C_T_ ~ β_0_ + β_1_*Condition + β_2_* C_T_[Gapdh] + β_3_* C_T_[beta-actin], where “Condition” referred to no injection or *Oct4* knockdown. C_T_ values were directly used in data analysis, as gene expression at the single-cell level has been shown to follow a lognormal distribution (6, 7).

References.

1. Li C & Wong WH (2003) in *The analysis of gene expression data: methods and software.* (Springer, New York), pp. 120-141.

2. Ashburner M*, et al.* (2000) Gene ontology: tool for the unification of biology. The Gene Ontology Consortium. *Nat Genet* 25, 25-29.

3. Beissbarth T & Speed TP (2004) GOstat: find statistically overrepresented Gene Ontologies within a group of genes. *Bioinformatics* 20, 1464-1465.

4. Zhou Q, Chipperfield H, Melton DA, & Wong WH (2007) A gene regulatory network in mouse embryonic stem cells. *Proc Natl Acad Sci U S A* 104, 16438-16443.

5. R.C. Gentleman VJC, D.M. Bates, B. Bolstad, M. Dettling, S. Dudoit, B. Ellis, L. Gautier, Y. Ge, J. Gentry, K. Hornik, T. Hothorn, W. Huber, S. Iacus, R. Irizarry, F. Leisch, C. Li, M. Maechler, A.J. Rossini, G. Sawitzki, C. Smith, G. Smyth, L. Tierney, J.Y.H. Yang, J. Zhang. (2004) Bioconductor: Open software development for computational biology and bioinformatics. *Genome Biology* 5, R80.

6. Bengtsson M, Stahlberg A, Rorsman P, & Kubista M (2005) Gene expression profiling in single cells from the pancreatic islets of Langerhans reveals lognormal distribution of mRNA levels. *Genome Res* 15, 1388-1392.

7. Warren L, Bryder D, Weissman IL, & Quake SR (2006) Transcription factor profiling in individual hematopoietic progenitors by digital RT-PCR. *Proc Natl Acad Sci U S A* 103, 17807-17812.
