# Supplementary figures and images for: A Novel and Critical Role for Oct4 as a Regulator of the Maternal-Embryonic Transition
Source: PLoS One. 2008 Dec 31;3(12):e4109. doi: 10.1371/journal.pone.0004109 (PMC2614881; doi:10.1371/journal.pone.0004109)

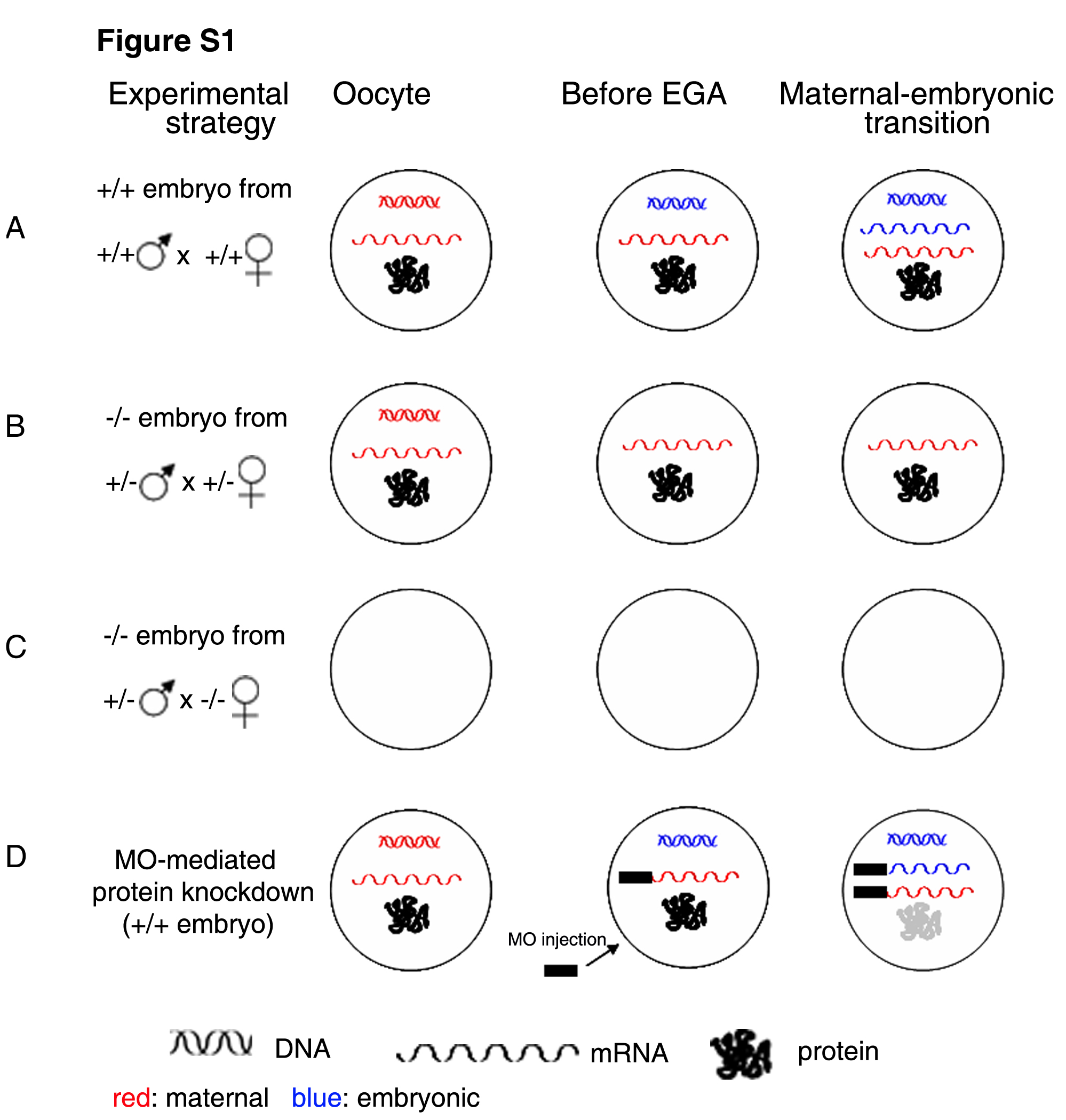

Supplement: Figure S1 — Limitation of conventional gene-targeting strategies for the study of gene function during the maternal-embryonic transition. Our overall goal is to address the specific functions of a given gene product at the maternal-embryonic transition in order to understand mechanisms that regulate mammalian embryo development at the earliest stages. In wild type (+/−) embryos, maternal transcripts are present before embryonic genome activation (EGA), while maternal and embryonic transcripts are present at maternal-embryonic transition stage, both resulting in production of a normal gene product (A). In homozygous null mutant (−/−) embryos generated from a mother that is heterozygous (+/−) for the null mutation, persistent maternal transcripts and/or proteins may “rescue” or delay the phenotype onset (B). In contrast, homozygous null mutant embryos generated from a homozygous mutant female, or a female with oocyte-specific gene deletion, the observed defects may reflect oocyte defects, rather than specific gene requirement in the early embryo (C). Therefore, these strategies do not address the precise roles of specific genes at the cusp of EGA or during EGA, when both maternal and early embryonic transcripts may be present simultaneously. Cytoplasmic microinjection of antisense morpholino oligonucleotides (MOs) into wild type embryo just at or before EGA results in specific translational block of both maternal and embryonic gene transcripts (D). Since MOs persist for at least a few cycles of cell division, gene-specific translational block is presumably effective until the morula-blastocyst stages (1–3). The absence of gene product during these developmental stages would reveal critical gene function and unmask early phenotypes that may not be detectable in conventional gene-targeting strategies by homologous recombination and transgenesis. While this model is well established in other species (2, 4, 5), it shifts the paradigm from investigating function of embryonic genes to [file pone.0004109.s002.doc]

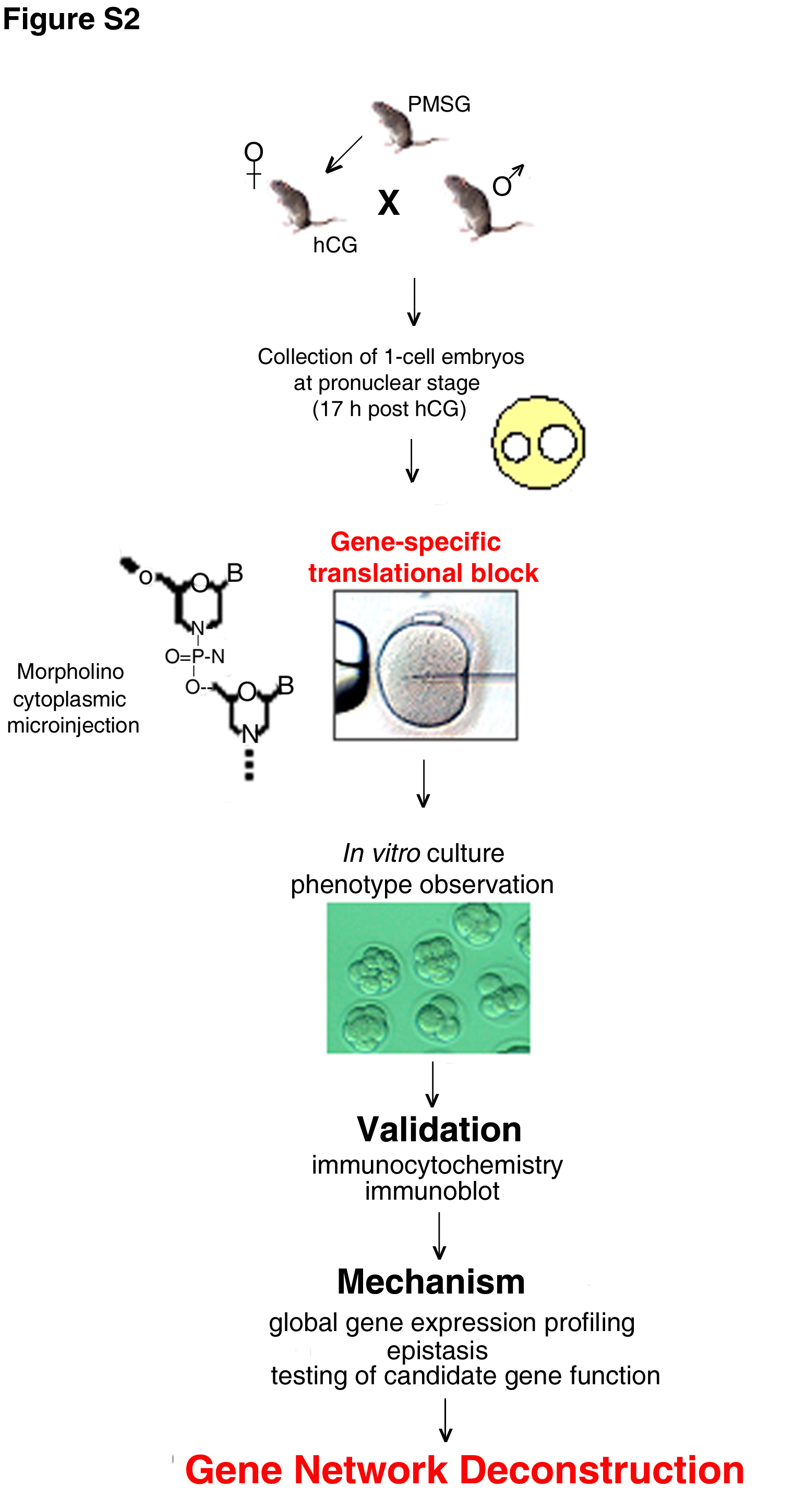

Supplement: Figure S2 — Experimental strategy. Embryos at the 2 pronuclei (2PN) or 1-cell stage are collected from wild type matings, and injected with an antisense morpholino oligomer (MO) that has been designed to target a specific gene. MO binds to 5′ UTR or transcription start site and blocks translation by steric hindrance. Microinjected embryos and uninjected control embryos are cultured in vitro and observed for developmental phenotypes such as fragmentation, or arrest at the 2-cell, 4-cell, multicell, or morula stages. Theoretically, this strategy may uncover other phenotypes such as asymmetrical division, but we have not observed them in the genes that we have tested. If a gene-specific MO produces the same phenotype consistently, while the mismatch control MO allows normal development, then we validate knockdown of the gene of interest by immunocytochemistry and/or immunoblotting. Mechanism of gene function is further investigated by obtaining global gene expression profiles from injected and control embryos at the mid-2-cell stage (43 hours post-HCG). Candidate downstream genes are tested for differential expression, and gene function in the early embryo. It is expected that multiple iterations of this strategy to test functions of different transcriptional regulators and their downstream targets will help to deconstruct the gene regulatory network in the mouse embryo at the cusp of embryonic genome activation. (0.77 MB DOC) [file pone.0004109.s003.doc]

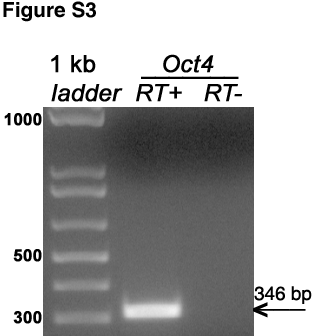

Supplement: Figure S3 — Oct4 expression in the mouse zygote by single embryo RT-PCR. (0.11 MB TIF) [file pone.0004109.s004.tif]

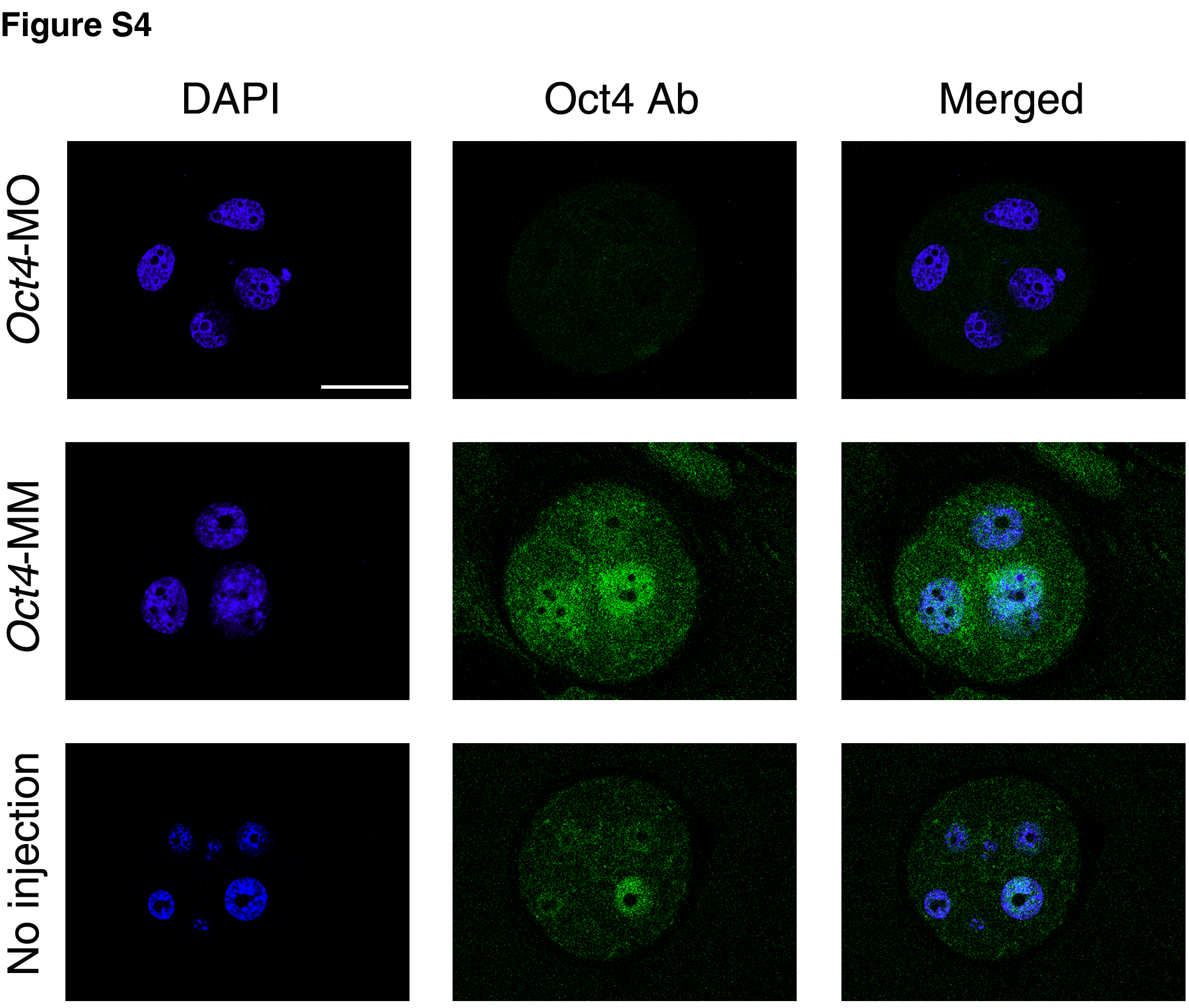

Supplement: Figure S4 — Decreased Oct4 expression was evident by the 4-cell stage in Oct4-MO-injected embryos. Oct4 signal was absent in embryos injected with Oct4-MO, but its nuclear localization was present in uninjected and mismatch controls. Scale bar 40 µm. (10.94 MB TIF) [file pone.0004109.s005.tif]

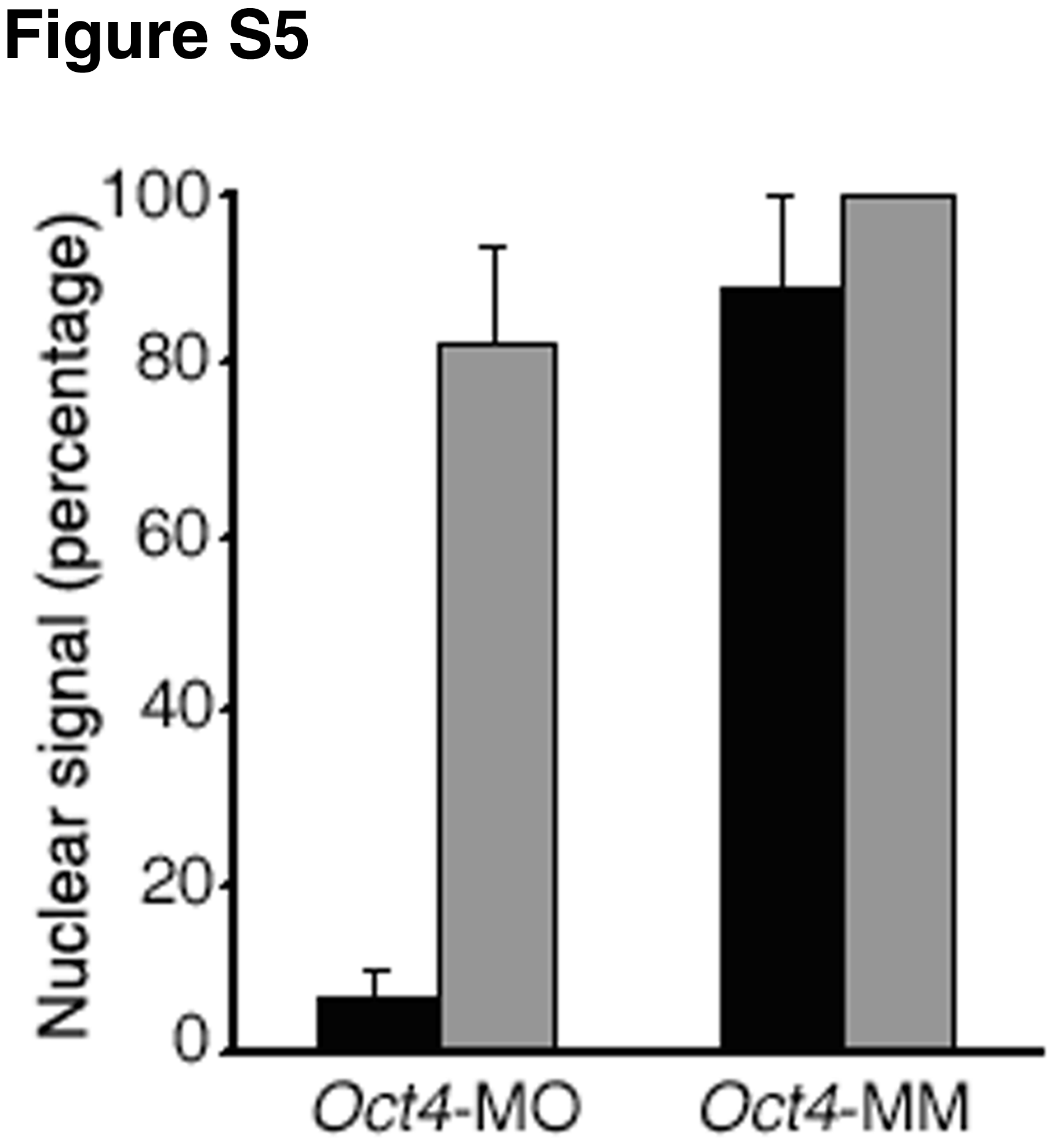

Supplement: Figure S5 — Decreased Oct4 expression at the multicell stage in Oct4-MO-injected embryos. Only 6.4±3.2% of Oct4-MO-injected embryos showed nuclear Oct4 signal, while 88.9±11.1% of Oct4-MM-injected embryos and 82.7±10.9% of uninjected control embryos showed unequivocal nuclear Oct4 expression at the multicell stage; p<0.05. (10.82 MB TIF) [file pone.0004109.s006.tif]

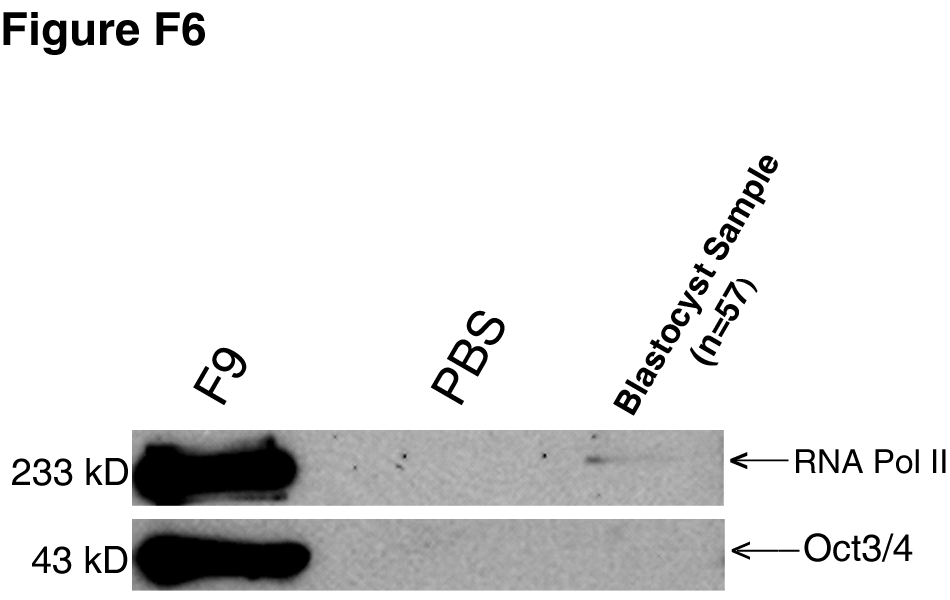

Supplement: Figure S6 — Oct4 knockdown could not be assessed by western blot. Anti-Oct4 antibody detected specific Oct4 band in F9 mouse embryonal carcinoma cell line, but not in the lane containing pooled protein lysate from 57 mouse blastocysts. In contrast, the band corresponding to RNA polymerase II (subunit A) was detectable in both F9 cells and mouse blastocysts. This figure is representative of experiments showing that 50–100 embryos at the 2-cell, multicell, and blastocyst stages did not have sufficient amounts of Oct4 protein for detection by western blot. (PBS, phosphate buffered saline) (0.57 MB TIF) [file pone.0004109.s007.tif]

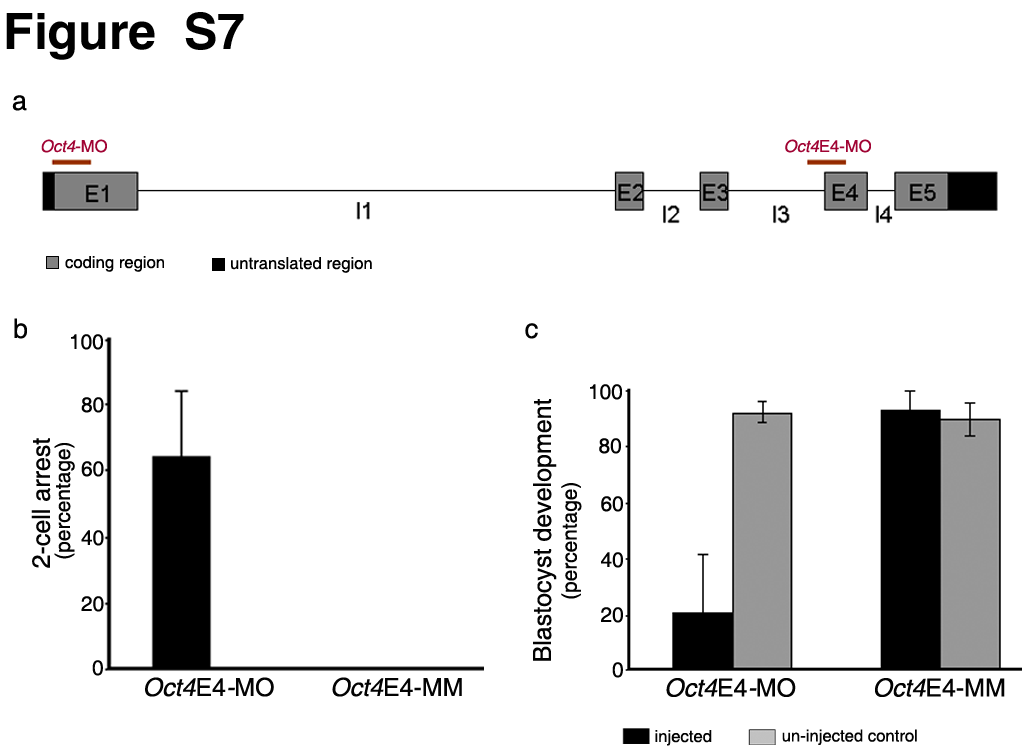

Supplement: Figure S7 — Confirmation of the requirement of Oct4 in early embryo development by Oct4E4-MO, an antisense morpholino that targets the splice site of exon 4 of Oct4. a, sites targeted by the two morpholinos, Oct4-MO and Oct4E4-MO. Oct4-MO targets the 25 nucleotides starting at the ATG start site, while Oct4E4-MO targets the splice site at the intron (I)-exon (E) boundary of the 4th exon (E4). Removal of E4 is expected to result in a protein product that lacks the DNA-binding and activation domains (1). b, 64.6±19.9% of embryos injected with Oct4E4-MO, while none that were injected with the mismatch control, Oct4E4-MM, arrested at the 2-cell stage. c, Blastocyst development is severely compromised after injection of Oct4E4-MO compared to the mismatch control, Oct4E4-MM. Reference. 1. Morcos PA (2007) Achieving targeted and quantifiable alteration of mRNA splicing with Morpholino oligos. Biochem Biophys Res Commun 358, 521–527. (2.33 MB TIF) [file pone.0004109.s008.tif]
