# Supplementary material for: A Novel and Critical Role for Oct4 as a Regulator of the Maternal-Embryonic Transition
Source: PLoS One. 2008 Dec 31;3(12):e4109. doi: 10.1371/journal.pone.0004109 (PMC2614881; doi:10.1371/journal.pone.0004109)
Supplement: Table S1 — Antisense morpholino oligonucleotides target gene-specific sequence in the 5′UTR and/or start site. (0.06 MB PDF) [file pone.0004109.s009.pdf]

Table S1. Antisense morpholino oligonucleotides target gene-specific sequence in the 5'UTR and/or start site. (Mismatched nucleotides are underlined.)

Name	Sequence	GC content, %
Ccna2-MO-1	5′-TCGAGGTGCCCGGCATCGCGGCTCC-3′	76
Ccna2-MO-2	5′-CTGTCGGCGGCAGAGCGTTCACAGC-3′	68
Ccna2-MM-1	5′-TCCAGGTCCCCCGCATCCCGGATCC-3′	72
Oct4-MO	5′-AGTCTGAAGCCAGGTGTCCAGCCAT-3′	56
Oct4-MM	5′-ACTCTCAAGCCACGTGTGCAGCGAT-3′	56
*Oct4*E4-MO	5′-CTCCGATTTGCATATCTGGGCAGGG-3′	56
*Oct4*E4-MM	5′-CTGCGATTTCCATATGTGCGCACGG-3′	56
Standard control*	5′-TCCAGGTCCCCCGCATCCCGGATCC-3′	72

* splice site of mutated human β-globin gene
