# Supplementary material for: A Novel and Critical Role for Oct4 as a Regulator of the Maternal-Embryonic Transition
Source: PLoS One. 2008 Dec 31;3(12):e4109. doi: 10.1371/journal.pone.0004109 (PMC2614881; doi:10.1371/journal.pone.0004109)
Supplement: Table S2 — Summary of the number of embryos tested and the number of experiments performed for each condition. (0.07 MB PDF) [file pone.0004109.s010.pdf]

Table S2. Summary of the number of embryos tested and the number of experiments performed for each condition.

Experiment	Condition	No. Injected Embryos	No. Uninjected Embryos	No. experiments
Ccna2 knockdown	*Ccna2*-MO 0.75 mM	94	65	8
	Ccna2-MM 0.75 mM	75	101	9
Ccna2-MO Immunocytochemistry	*Ccna2*-MO 0.75 mM	26	28	3
Ccna2 “gene dosage”	*Ccna2*-MO, 0.5 mM	39	30	3
	Ccna2-MO, 0.25 mM	50	29	3
Oct4 knockdown	*Oct4*-MO, 0.60 mM	41	37	4
	Oct4-MM, 0.60 mM	57	30	3
	Oct4-MO, 0.40 mM	79	25	3
	Oct4-MO, 0.20 mM	63	30	3
	*Oct4*E4-MO, 0.60 mM	35	30	3
	*Oct4*E4-MM, 0.60 mM	32	30	3
Oct4-MO Immunocytochemistry	*Oct4*-MO, 0.60 mM	32	22	3
	Oct4-MM, 0.60 mM	20	20	3
Rescue	0.036µg/µl *Oct4* mRNA+ 0.60mM *Oct4*-MO	32	30	3
	0.036µg/µl *EYFP* mRNA + 0.60mM *Oct4*-MO	72	30	3
Overexpression	0.09µg/µl *Oct4* mRNA	24	20	3
	0.036µg/µl *Oct4* mRNA	33	25	3
	3.6ng/µl *Oct4* mRNA	35	30	3
	0.09µg/µl *EYFP* mRNA	41	20	2
Oct4-MO/Conditioned Media	*Oct4*-MO, 0.60 mM, conditioned media	20	20	2
	Oct4-MO, 0.60 mM, unconditioned media	20	20	2
Ccna2-MO gene chip	*Ccna2*-MO, 0.75 mM	60	60	3
Oct4-MO gene chip	*Oct4*-MO, 0.6 mM	60	60	3
Oct4-MO single embryo QPCR	*Oct4*-MO, 0.60 mM, 2-cell stage	14-20	14-20	3-5
	Control-MO, 0.60 mM, 2-cell stage	5-10	n/a	2
