# Supplementary material for: A Novel and Critical Role for Oct4 as a Regulator of the Maternal-Embryonic Transition
Source: PLoS One. 2008 Dec 31;3(12):e4109. doi: 10.1371/journal.pone.0004109 (PMC2614881; doi:10.1371/journal.pone.0004109)
Supplement: Table S7 — Oct4 candidate target genes that have putative Oct4 binding sites based on genomic sequence analysis or mouse ESC chromatin precipitation data*. *Data were compared to those reported in: Zhou Q, Chipperfield H, Melton DA, & Wong WH (2007) A gene regulatory network in mouse embryonic stem cells. Proc Natl Acad Sci U S A 104, 16438–16443. (0.02 MB PDF) [file pone.0004109.s015.pdf]

Table S7. Oct4 candidate target genes that have putative Oct4 binding sites based on genomic sequence analysis or mouse ESC chromatin precipitation (ChIP) data(4).

Downregulated in *Oct4* knockdown embryos:
1700021F05Rik: RIKEN cDNA 1700021F05 gene
2900073H19Rik: RIKEN cDNA 2900073H19 gene
Arhgap8: Rho GTPase activating protein 8
Cbfa2t2h: core-binding factor, runt domain, alpha subunit 2, translocated to, 2 homolog (human)
Cdt1: chromatin licensing and DNA replication factor 1
Dido1: death inducer-obliterator 1
Dpm1: dolichol-phosphate (beta-D) mannosyltransferase 1
Dppa5: developmental pluripotency associated 5
Eif4e2: eukaryotic translation initiation factor 4E member 2
Elovl6: ELOVL family member 6, elongation of long chain fatty acids (yeast)
Etv5: ets variant gene 5
Fkbp4: FK506 binding protein 4
Gnb2l1: guanine nucleotide binding protein (G protein), beta polypeptide 2 like 1
Hexb: hexosaminidase B
Igf2bp1: insulin-like growth factor 2 mRNA binding protein 1
Klf9: Kruppel-like factor 9
Mkrn1: makorin, ring finger protein, 1
Mtf2: metal response element binding transcription factor 2
Myg1: melanocyte proliferating gene 1
Pa2g4: proliferation-associated 2G4
Pitpnc1: phosphatidylinositol transfer protein, cytoplasmic 1
Ppm1a: protein phosphatase 1A, magnesium dependent, alpha isoform
Rest: RE1-silencing transcription factor
Rif1 /// LOC671598: Rap1 interacting factor 1 homolog (yeast) /// similar to Telomere-associated protein RIF1 (Rap1-interacting factor 1 homolog) (mRif1)
Slc19a3: solute carrier family 19 (sodium/hydrogen exchanger), member 3
Slc22a12: solute carrier family 22 (organic anion/cation transporter), member 12
Slc25a36: solute carrier family 25, member 36
Tfrc: transferrin receptor
Tnpo3: transportin 3
Ube2h: ubiquitin-conjugating enzyme E2H
Ube2o: ubiquitin-conjugating enzyme E2O
Zfp297b: zinc finger protein 297B

Upregulated in *Oct4* knockdown embryos
BC022623: cDNA sequence BC022623
2810429O05Rik: RIKEN cDNA 2810429O05 gene
Blcap: bladder cancer associated protein homolog (human)
Icosl: icos ligand
Sox2: SRY-box containing gene 2
Bcas2: breast carcinoma amplified sequence 2
Ldlr: low density lipoprotein receptor
Zfp219: zinc finger protein 219
Arl4c /// LOC632433: ADP-ribosylation factor-like 4C /// similar to ADP-ribosylation factor-like protein 7
Tcl1: T-cell lymphoma breakpoint 1
Dcp1a: decapping enzyme
Nes: nestin
Rbpsuh: Recombining binding protein suppressor of hairless (Drosophila)
