# Supplementary material for: A Novel and Critical Role for Oct4 as a Regulator of the Maternal-Embryonic Transition
Source: PLoS One. 2008 Dec 31;3(12):e4109. doi: 10.1371/journal.pone.0004109 (PMC2614881; doi:10.1371/journal.pone.0004109)
Supplement: Table S8 — Functional categories that were enriched in downregulated genes in the Ccna2 knockdown model. (0.03 MB PDF) [file pone.0004109.s016.pdf]

Table S8. Functional categories that were enriched in downregulated genes in the *Ccna2* knockdown model.

	GOBPID	P-value	Term
1	GO:0050875	1.1E-3	cellular physiological process
2	GO:0016568	4.0E-3	chromatin modification*
3	GO:0042254	4.0E-3	ribosome biogenesis and assembly
4	GO:0006338	4.8E-3	chromatin remodeling*
5	GO:0007028	0.01	cytoplasm organization and biogenesis
6	GO:0044237	0.01	cellular metabolism
7	GO:0007582	0.01	physiological process
8	GO:0006139	0.01	nucleobase, nucleoside, nucleotide and nucleic acid metabolism*
9	GO:0008152	0.01	metabolism
10	GO:0006376	0.01	mRNA splice site selection*
11	GO:0006413	0.01	translational initiation
12	GO:0007001<br>GO:0007001<br>[illegible]	0.01	chromosome organization and biogenesis (sensu Eukaryota)*
13	GO:0006996	0.01	organelle organization and biogenesis*
21	GO:0007046	0.01	ribosome biogenesis
22	GO:0043170	0.02	macromolecule metabolism
23	GO:0051276	0.02	chromosome organization and biogenesis
24	GO:0006325	0.02	establishment and/or maintenance of chromatin
25	GO:0006323	0.02	DNA packaging*
26	GO:0016043	0.02	cell organization and biogenesis
27	GO:0044238	0.03	primary metabolism
28	GO:0000245	0.03	spliceosome assembly
42	GO:0006486	0.03	protein amino acid glycosylation
43	GO:0009059	0.03	macromolecule biosynthesis
44	GO:0043413	0.03	biopolymer glycosylation
45	GO:0009101	0.04	glycoprotein biosynthesis
57	GO:0006364	0.04	rRNA processing
58	GO:0006412	0.04	protein biosynthesis
59	GO:0006259	0.04	DNA metabolism
60	GO:0016072	0.04	rRNA metabolism
61	GO:0009100	0.05	glycoprotein metabolism

Note: Highlighted categories were enriched in the *Oct4* data set as well. Asterisk indicates functional categories that were specifically enriched in *Ccna2* knockdown.
