# Supplementary material for: A Novel and Critical Role for Oct4 as a Regulator of the Maternal-Embryonic Transition
Source: PLoS One. 2008 Dec 31;3(12):e4109. doi: 10.1371/journal.pone.0004109 (PMC2614881; doi:10.1371/journal.pone.0004109)
Supplement: Table S9 — Functional categories that were enriched in upregulated genes in the Ccna2 knockdown model. (0.01 MB PDF) [file pone.0004109.s017.pdf]

Table S9. Functional categories that were enriched in upregulated genes in the *Ccna2* knockdown model.

	GOBPID	P-value	Term
1	GO:0044262	7.1E-4	cellular carbohydrate metabolism
