# Supplementary material for: A Novel and Critical Role for Oct4 as a Regulator of the Maternal-Embryonic Transition
Source: PLoS One. 2008 Dec 31;3(12):e4109. doi: 10.1371/journal.pone.0004109 (PMC2614881; doi:10.1371/journal.pone.0004109)
Supplement: Table S10 — Functional categories that were enriched in downregulated genes in the Oct4 knockdown model. (0.02 MB PDF) [file pone.0004109.s018.pdf]

Table S10. Functional categories that were enriched in downregulated genes in the *Oct4* knockdown model.

	GOBPID	P-value	Term
1	GO:0006412	1.8E-7	protein biosynthesis
2	GO:0009059	1.3E-6	macromolecule biosynthesis
3	GO:0006413	3.6E-6	translational initiation
4	GO:0009058	1.8E-5	biosynthesis
5	GO:0044249	2.1E-5	cellular biosynthesis
6	GO:0042254	2.3E-5	ribosome biogenesis and assembly
7	GO:0006396	3.0E-5	RNA processing*
8	GO:0007046	3.3E-5	ribosome biogenesis
9	GO:0016070	3.9E-5	RNA metabolism
10	GO:0007028	7.0E-5	cytoplasm organization and biogenesis
11	GO:0043037	1.1E-4	Translation*
12	GO:0043170	1.2E-4	macromolecule metabolism
13	GO:0019538	1.6E-4	protein metabolism
14	GO:0044238	3.1E-4	primary metabolism
15	GO:0044267	3.1E-4	cellular protein metabolism
16	GO:0050875	3.3E-4	cellular physiological process
17	GO:0044237	5.3E-4	cellular metabolism
18	GO:0044260	5.7E-4	cellular macromolecule metabolism

Note: Highlighted categories were enriched in the *Ccna2* data set as well. Asterisk indicates functional categories that were specifically enriched in *Oct4* knockdown.
