# Supplementary material for: A Novel and Critical Role for Oct4 as a Regulator of the Maternal-Embryonic Transition
Source: PLoS One. 2008 Dec 31;3(12):e4109. doi: 10.1371/journal.pone.0004109 (PMC2614881; doi:10.1371/journal.pone.0004109)
Supplement: Table S11 — Functional categories that were enriched in upregulated genes in the Oct4 knockdown model. (0.01 MB PDF) [file pone.0004109.s019.pdf]

Table S11. Functional categories that were enriched in upregulated genes in the *Oct4* knockdown model.

	GOBPID	P-value	Term
1	GO:0044262	2.0E-4	Alcohol metabolism
