# Supplementary material for: A Novel and Critical Role for Oct4 as a Regulator of the Maternal-Embryonic Transition
Source: PLoS One. 2008 Dec 31;3(12):e4109. doi: 10.1371/journal.pone.0004109 (PMC2614881; doi:10.1371/journal.pone.0004109)
Supplement: Table S12 — Some candidate Oct4-regulated genes that function in transcription, translation, RNA processing, chromatin remodeling, signaling, apoptosis and the cell cycle. (0.08 MB PDF) [file pone.0004109.s020.pdf]

Table S12. Some candidate Oct4-regulated genes that function in transcription, translation, RNA processing, chromatin remodeling, signaling, apoptosis and the cell cycle.

	Gene Symbol	Fold change	False detection rate (FDR)	T. P-value
**POL I transcription**				
upstream binding transcription factor, RNA polymerase I	Ubtf	0.49	0.02667	0.00424
arginyl-tRNA synthetase	Rars	0.47	0.03200	0.00131
**POL II transcription**				
BTAF1 RNA polymerase II, B-TFIID transcription factor-associated, (Mot1 homolog, S. cerevisiae)	Btaf1	0.12	0.03834	0.00833
mediator of RNA polymerase II transcription, subunit 8 homolog (yeast)	Med8	0.25	0.03405	0.00142
transcription elongation factor B (SIII), polypeptide 3	Tceb3	0.29	0.01471	0.00001
TAF9 RNA polymerase II, TATA box binding protein (TBP)-associated factor	Taf9	0.30	0.02151	0.00027
polymerase (RNA) II (DNA directed) polypeptide H	Polr2h	0.42	0.02586	0.00859
elongation factor RNA polymerase II 2	Ell2	0.49	0.03696	0.00098
cofactor required for Sp1 transcriptional activation, subunit 2	Med14	0.50	0.03794	0.00156
**POL III transcription**				
general transcription factor IIIC, polypeptide 4	Gtf3c4	0.07	0.00000	0.00002
general transcription factor IIIC, polypeptide 5	Gtf3c5	0.34	0.03209	0.00212
BRF2, subunit of RNA polymerase III transcription initiation factor, BRF1-like	Brf2	0.34	0.04385	0.05104
general transcription factor IIIC, polypeptide 2, beta	Gtf3c2	0.35	0.03314	0.00047
general transcription factor III C 1	Gtf3c1	0.47	0.03303	0.00236
polymerase (RNA) III (DNA directed) polypeptide A	Polr3a	0.55	0.04421	0.01694
polymerase (RNA) III (DNA directed) polypeptide E	Polr3e	0.56	0.03366	0.00134
**Translational control, RNA processing, and posttranscriptional regulation**				
**Translational initiation**				
eukaryotic translation initiation factor 2C, 5	Eif2c5	0.09	0.01370	0.00022
eukaryotic translation initiation factor 5B	Eif5b	0.14	0.03802	0.00002
eukaryotic translation initiation factor 3, subunit 9 (eta)	Eif3b	0.29	0.03803	0.00358
eukaryotic translation initiation factor 3, subunit 8	Eif3c	0.36	0.00000	0.00016
eukaryotic translation initiation factor 3, subunit 3 (gamma)	Eif3h	0.42	0.03226	0.00485
eukaryotic translation initiation factor 3, subunit 4 (delta)	Eif3g	0.47	0.04924	0.01507
integrin beta 4 binding protein	Eif6	0.53	0.02756	0.00236
eukaryotic translation initiation factor 4, gamma 1	Eif4g1	0.59	0.04065	0.00041
eukaryotic translation initiation factor 2, subunit 1 alpha	Eif2s1	0.60	0.04879	0.00311
eukaryotic translation initiation factor 4E /// hypothetical LOC630527	Eif4e	0.65	0.03742	0.00177
eukaryotic translation initiation factor 3, subunit 10 (theta)	Eif3s10	0.66	0.04070	0.01798
**Translational repression**				
piwi-like homolog 2 (Drosophila)	Piwil2	0.34	0.02778	0.00051
eukaryotic translation initiation factor 4E member 2	Eif4e2	0.46	0.02553	0.01084

**3' end processing**				
poly (A) polymerase alpha	Papola	0.10	0.02475	0.00014
cleavage and polyadenylation specific factor 6	Cpsf6	0.20	0.03256	0.00009
cleavage and polyadenylation specific factor 4	Cpsf4	0.48	0.03542	0.00233
**Translational termination**				
eukaryotic translation termination factor 1	Etf1	0.36	0.02717	0.00207
**Nonsense mediated decay (NMD)**				
eukaryotic translation initiation factor 3, subunit 6	Eif3e	0.21	0.00000	0.00000
**Other RNA binding and processing**				
integrator complex subunit 7	Ints7	0.10	0.02757	0.00004
RNA binding motif protein 3	Rbm3	0.20	0.03791	0.00037
RNA binding motif protein 4	Rbm4	0.42	0.04967	0.00236
developmental pluripotency associated 5	Dppa5	0.46	0.03672	0.00786
RNA binding motif protein 5	Rbm5	0.52	0.03415	0.00245
proliferation-associated 2G4	Pa2g4	0.70	0.04956	0.01729
**Pre-mRNA processing and transport**				
Heterogeneous nuclear ribonucleoprotein U		0.18	0.03688	0.00020
PRP4 pre-mRNA processing factor 4 homolog (yeast)	Prpf4	0.34	0.03828	0.00128
RNA, U transporter 1	Snupn	0.35	0.01681	0.00227
PRP38 pre-mRNA processing factor 38 (yeast) domain containing B	Prpf38b	0.48	0.04103	0.00548
protein phosphatase 1G (formerly 2C), magnesium-dependent, gamma isoform	Ppm1g	0.48	0.02463	0.00012
heterogeneous nuclear ribonucleoprotein A/B	Hnrpab	0.58	0.03233	0.00100
heterogeneous nuclear ribonucleoprotein A1	Hnrpa1	0.63	0.04981	0.00961
heterogeneous nuclear ribonucleoprotein K	Hnrpk	0.64	0.04473	0.01239
heterogeneous nuclear ribonucleoprotein M	Hnrpm	0.63	0.04884	0.00724
**Transcriptional regulation**				
homeodomain interacting protein kinase 3	Hipk3	0.17	0.04003	0.00031
Kruppel-like factor 9	Klf9	0.17	0.00000	0.00020
nuclear respiratory factor 1	Nrf1	0.20	0.01923	0.00073
integrator complex subunit 4	Ints4	0.34	0.03520	0.00080
homeodomain interacting protein kinase 1	Hipk1	0.35	0.01786	0.00073
Pbx/knotted 1 homeobox	Pknox1	0.37	0.03796	0.00484
proline, glutamic acid and leucine rich protein 1	Pelp1	0.40	0.02415	0.00573
core-binding factor, runt domain, alpha subunit 2, translocated to, 2 homolog (human)		0.41	0.04076	0.02903
YY1 transcription factor	Yy1	0.43	0.03853	0.00074
GATA binding protein 4	Gata4	0.45	0.03963	0.00325
metastasis-associated gene family, member 2	Mta2	0.51	0.03395	0.00326
BCL2-associated transcription factor 1	Bclaf1	0.54	0.02664	0.00242
RE1-silencing transcription factor	Rest	0.62	0.03827	0.00983
ets variant gene 5	Etv5	0.50	0.04900	0.00576
makorin, ring finger protein, 1	Mkrn1	0.58	0.03793	0.00310
**Chromatin remodeling, epigenetic regulation**				
jumonji domain containing 1B	Kdm3b	0.14	0.03770	0.00132
jumonji, AT rich interactive domain 1C (Rbp2 like)	Kdm5c	0.23	0.01408	0.00075
sirtuin 1 ((silent mating type information regulation 2, homolog) 1 (S. cerevisiae)	Sirt1	0.28	0.01136	0.00002
methyltransferase like 2	Mettl2	0.37	0.03333	0.00010

jumonji domain containing 1A	Kdm3a	0.38	0.04874	0.00764
SWI/SNF related, matrix associated, actin dependent regulator of chromatin, subfamily c, member 1	Rsc8	0.41	0.03665	0.01049
jumonji, AT rich interactive domain 1B (Rbp2 like)	Kdm5b	0.57	0.03738	0.00388
eukaryotic translation initiation factor 2, subunit 3, structural gene X-linked /// similar to eukaryotic translation initiation factor 2, subunit 3, structural gene X-linked		0.57	0.04905	0.00183
**Histone H3-specific HAT activity**				
general transcription factor IIIC, polypeptide 4	Gtf3c4	0.07	0.00000	0.00002
general transcription factor IIIC, polypeptide 5	Gtf3c5	0.34	0.03209	0.00212
GCN5 general control of amino acid synthesis-like 2 (yeast)	Gcn5	0.34	0.02525	0.00159
general transcription factor IIIC, polypeptide 2, beta	Gtf3c2	0.35	0.03314	0.00047
general transcription factor III C 1	Gtf3c1	0.47	0.03303	0.00236
H3 histone, family 3A	H3f3a	0.53	0.04006	0.00865
**Signaling pathways (Fgf4, Bmp, Toll, Egf, Mapk, Erk, Igf2, Amp)**				
fibroblast growth factor 4	Fgf4	0.09	0.02591	0.00088
signaling intermediate in Toll pathway-evolutionarily conserved	Ecsit	0.17	0.01935	0.00000
protein kinase, AMP-activated, gamma 1 non-catalytic subunit	Prkag1	0.19	0.03786	0.00010
insulin-like growth factor 2 mRNA binding protein 1	Imp1	0.26	0.04930	0.00047
bone morphogenetic protein receptor, type 1A	Bmpr1a	0.35	0.02347	0.00005
fibroblast growth factor receptor-like 1	Fgfrl1	0.34	0.03349	0.01815
interleukin 17 receptor D	Il17rd	0.35	0.01961	0.00052
mitogen activated protein kinase 1	Mapk1	0.39	0.04889	0.00710
splicing factor, arginine/serine-rich 15 /// similar to splicing factor, arginine/serine-rich 15	Sfrs15	0.47	0.02113	0.00047
protein phosphatase 1A, magnesium dependent, alpha isoform	Ppm1a	0.46	0.02525	0.00280
protein kinase C, delta	Prkcd	0.45	0.03465	0.02184
insulin-like growth factor 2 receptor	Igf2r	0.53	0.04738	0.00600
**Apoptosis**				
junction-mediating and regulatory protein	Jmy	0.21	0.00000	0.00036
sphingosine-1-phosphate phosphatase 1	Sgpp1	0.23	0.01538	0.00160
cathepsin B	Ctsb	0.28	0.00000	0.00024
death inducer-obliterator 1	Dido1	0.46	0.03440	0.00196
**Cell cycle, DNA replication, DNA repair, cell division, chromosome segregation, cell growth, cell size**				
origin recognition complex, subunit 4-like (S. cerevisiae)	Orc4l	0.17	0.01852	0.00383
purine rich element binding protein B	Purb	0.29	0.02669	0.00019
chromosome segregation 1-like (S. cerevisiae)	Cse1l	0.34	0.03254	0.00002
minichromosome maintenance deficient 5, cell division cycle 46 (S. cerevisiae)	Mcm5	0.34	0.01852	0.00004
kinesin family member 11	Kif11	0.34	0.01938	0.00170
replication factor C (activator 1) 2	Rfc3	0.40	0.04420	0.00919
kinesin family member 22	Kif22	0.42	0.02667	0.00033
protein phosphatase 2 (formerly 2A), catalytic subunit, beta isoform	Ppp2cb	0.43	0.03243	0.00658
centromere protein E	Cenpe	0.54	0.03801	0.00299
Rap1 interacting factor 1 homolog (yeast) /// similar to Telomere-associated protein RIF1 (Rap1-interacting factor 1 homolog) (mRif1)	mRif1	0.63	0.04515	0.00228
