# Supplementary material for: A Novel and Critical Role for Oct4 as a Regulator of the Maternal-Embryonic Transition
Source: PLoS One. 2008 Dec 31;3(12):e4109. doi: 10.1371/journal.pone.0004109 (PMC2614881; doi:10.1371/journal.pone.0004109)
Supplement: Table S13 — Gene-specific primers and Taqman® probes that were used in q-PCR experiments. (0.04 MB PDF) [file pone.0004109.s021.pdf]

Table S13. Gene-specific primers and Taqman® probes that were used in q-PCR experiments.

Gene-specific primers used for Oct4 (302 bp product) were GGCGTTCTCTTTGGAAAGGTGTT (forward) and CTCGAACCACATCCTTCTCT (reverse), and those for *Ccna2* (212 bp product) were GATAGATTCCTCTCCTCCATG (forward) and TCACACACTTAGTGTCTCTGG (reverse).

Probe Name	Applied Biosystems Assay ID/ Catalog No.
Bmpr1a	Mm01208758_m1
Pou5f1	Mm00658129_gH
Klf9	Mm00495172_m1
Fgf4	Mm00438917_m1
Sall4	Mm01240680_m1
Bclaf1	Mm00464127_m1
Yy1	Mm00456392_m1
Pknox1	Mm00479320_m1
Gata4	Mm00484689_m1
Mta2	Mm00488671_m1
Rest1	Mm00803268_m1
Tcfl5	Mm00626495_m1
Dppa5	Mm01171664_g1
Bmpr1a	Mm00477650_m1
Fgfrl1	Mm00475318_g1
Il17rd	Mm00460340_m1
Ubtf	Mm00456972_m1
Gtf3c4	Mm00557022_m1
Gtf3c2	Mm00510828_m1
Polr3e	Mm00491765_m1
Polr3a	Mm00805896_m1
Eif3b	Mm00659801_m1
Eif3s10	Mm00468721_m1
Piwil2	Mm00502383_m1
Eif4e	Mm00725633_s1
Polr2h	Mm01344328_g1
Eif2c5	Mm01305462_m1
Eif3c	Mm01278697_m1
Eif5b	Mm01227234_m1
Papola	Mm01334253_m1
Eif3e	Mm01700222_g1
Sox2	Mm03053810_s1

Probe Name	Applied Biosystems Assay ID/ Catalog No.
Mouse b-actin	4352341E
Mouse b-actin	4352933E
Mouse Gapdh	4352932E
